# Heterologous Expression of *Saussurea involucrata SiPIP1;5b* Enhances Freezing Tolerance in *Arabidopsis* and Chilling Tolerance in Tomato

**DOI:** 10.3390/plants15203204

**Published:** 2026-10-19

**Authors:** Xiaoyan Wu, Saisai Wang, Bo Yin, Weixiang Zhou, Zhenhai Zhang, Hongliang Xin, Hongxiang Zhang, Bo Cui, Wenwen Xia, Ruina Liu, Jin Li, Jianbo Zhu

**Affiliations:** College of Life Science, Shihezi University, Shihezi 832000, China

**Keywords:** *Saussurea involucrata*, *SiPIP1;5b*, aquaporin, low-temperature tolerance, membrane integrity, reactive oxygen species, *Arabidopsis thaliana*, *Solanum lycopersicum*

## Abstract

Low temperature is a major constraint limiting the productivity and distribution of crops, and aquaporins have emerged as important determinants of plant stress tolerance. *Saussurea involucrata*, an alpine plant adapted to extreme low-temperature environments, provides a valuable source of cold-tolerance genes, yet the contribution of its PIP1-type plasma membrane aquaporins to freezing and chilling tolerance in heterologous systems has not been established. Here, we isolated *SiPIP1;5b*, a PIP1-type plasma membrane intrinsic protein from *S. involucrata*, and constitutively overexpressed it in *Arabidopsis thaliana* and tomato (*Solanum lycopersicum*). Under freezing treatment (−4 °C, 4 h), *SiPIP1;5b*-overexpressing *Arabidopsis* lines showed markedly higher survival rates (72.0–81.5%) than the wild type (36.2%), together with lower relative electrolyte leakage and malondialdehyde (MDA) content and higher chlorophyll content. Under chilling stress (0 °C, 4 h), transgenic tomato plants likewise exhibited reduced electrolyte leakage and MDA accumulation, elevated activities of superoxide dismutase, catalase, and peroxidase, and increased proline content relative to wild-type plants. These results indicate that *SiPIP1;5b* enhances freezing and chilling tolerance and is associated with improved membrane stability, reduced accumulation of excessive reactive oxygen species, and enhanced antioxidant and osmoprotective responses. Our findings establish *SiPIP1;5b* as a promising candidate gene for improving low-temperature tolerance in crops.

## 1. Introduction

Low temperature is a major environmental constraint that limits the geographical distribution of crops and reduces their productivity worldwide [1]. Freezing stress injures plants primarily by damaging the plasma membrane: extracellular ice crystals grow and impose cellular dehydration and hyperosmotic stress, and the resulting membrane lesions cause electrolyte leakage and, ultimately, cell death [2,3]. Plants that withstand freezing acquire tolerance through cold acclimation, which involves membrane remodeling, accumulation of protective osmolytes such as proline, and reinforcement of antioxidant defense [4,5]. Cold also triggers an overproduction of reactive oxygen species (ROS), which peroxidize membrane lipids and damage proteins and nucleic acids; their scavenging by enzymes such as superoxide dismutase (SOD), catalase (CAT), and peroxidase (POD) is therefore central to tolerance [1,6].

Beyond these biochemical responses, maintaining cellular water balance is critical for stress tolerance. Water movement across the plasma membrane is facilitated by aquaporins, a family of channel proteins that also transport small molecules such as CO_2_ and H_2_O_2_ [7,8]. Among them, the plasma membrane intrinsic proteins (PIPs) form the largest and most abundant subgroup and play a central role in plant water relations, stomatal movement, and hydraulic conductance [9,10]. On the basis of sequence features of their N- and C-termini, PIPs are divided into the PIP1 and PIP2 subfamilies; both share a characteristic topology of six transmembrane helices and two conserved asparagine–proline–alanine (NPA) motifs and can assemble into homo- or heterotetramers to regulate water permeability [11,12,13].

The expression and activity of PIPs are dynamically regulated by drought, salinity, and low temperature, indicating their involvement in stress adaptation [14,15,16]. Overexpression of several PIP genes improves stress tolerance: *AtPIP2;5* supports cold acclimation and freezing tolerance in *Arabidopsis* [17]; *OsPIP2;7* increases survival after freeze–thaw in rice [18]; *MfPIP2-7* from *Medicago falcata* confers cold tolerance in tobacco [19]; and *KoPIP2;1* from a mangrove enhances cold tolerance in *Arabidopsis* [20]. Notably, *CmPIP2;3* in melon links H_2_O_2_ signaling to antioxidative defense and cold tolerance [21], indicating that PIPs contribute to chilling tolerance beyond water transport. PIP1-type members have also been implicated in cold tolerance: overexpression of *AtPIP1;4* enhanced cold tolerance in *Arabidopsis* [22], and PIP1 aquaporins were among the early cold-induced proteins in the cold-tolerant banana cultivar Dajiao [23]. PIP1-type members, however, have received less attention than PIP2 in low-temperature studies, and their roles in freezing and chilling tolerance remain incompletely defined.

Extreme environments are a rich source of stress-tolerance genes. *Saussurea involucrata* Kar. et Kir., an alpine plant that grows at elevations of 2400–4100 m under persistent low temperature and strong ultraviolet radiation, has evolved a remarkable capacity to survive freezing and is regarded as an excellent model and gene pool for mining cold-tolerance genes [24,25]. Heterologous expression of *S. involucrata* genes, including the dehydrin *SiDHN* and the late embryogenesis abundant (LEA) proteins *SiLEA5* and *SiLEA B19.3*, has enhanced cold and drought tolerance in transgenic tomato [26,27,28]. Among the aquaporins of *S. involucrata*, the PIP1-type gene *SiPIP1;5a* is upregulated under low-temperature stress and associated with cold tolerance [29].

Despite these advances, the contribution of PIP1-type aquaporins from *S. involucrata* to freezing and chilling tolerance in heterologous systems has not been established. Tomato, a chilling-sensitive crop whose cold tolerance depends on functional CBF signaling [30], offers a practical target for such validation. Here, we cloned *SiPIP1;5b*, a PIP1-type plasma membrane aquaporin from *S. involucrata*, and constitutively expressed it in *Arabidopsis thaliana* and tomato (*Solanum lycopersicum*). We show that *SiPIP1;5b* overexpression enhances freezing tolerance in *Arabidopsis* and chilling tolerance in tomato, in association with maintenance of membrane stability, reduced ROS accumulation, elevated antioxidant enzyme activities, and increased proline content. Our results identify *SiPIP1;5b* as a promising candidate for improving low-temperature tolerance in crops.

## 2. Results

### 2.1. Sequence Features, Phylogenetic Position, and Structural Characteristics of SiPIP1;5b

The full-length coding sequence (CDS) of *SiPIP1;5b* was 882 bp in length. The 882-bp CDS contains 293 amino-acid-encoding codons plus one stop codon, and encodes a predicted protein of 293 amino acids. The predicted SiPIP1;5b protein had a molecular weight of 31.236 kDa and a theoretical isoelectric point (pI) of 8.54. The calculated net charge at pH 7.0 was 4.43. The predicted extinction coefficient at 280 nm was 44,140 M^−1^ cm^−1^, corresponding to an absorbance (A_280_) of approximately 1.41 for a 1 mg mL^−1^ protein solution. These physicochemical properties are consistent with *SiPIP1;5b* being a relatively small and slightly basic membrane protein.

**Figure 1:**
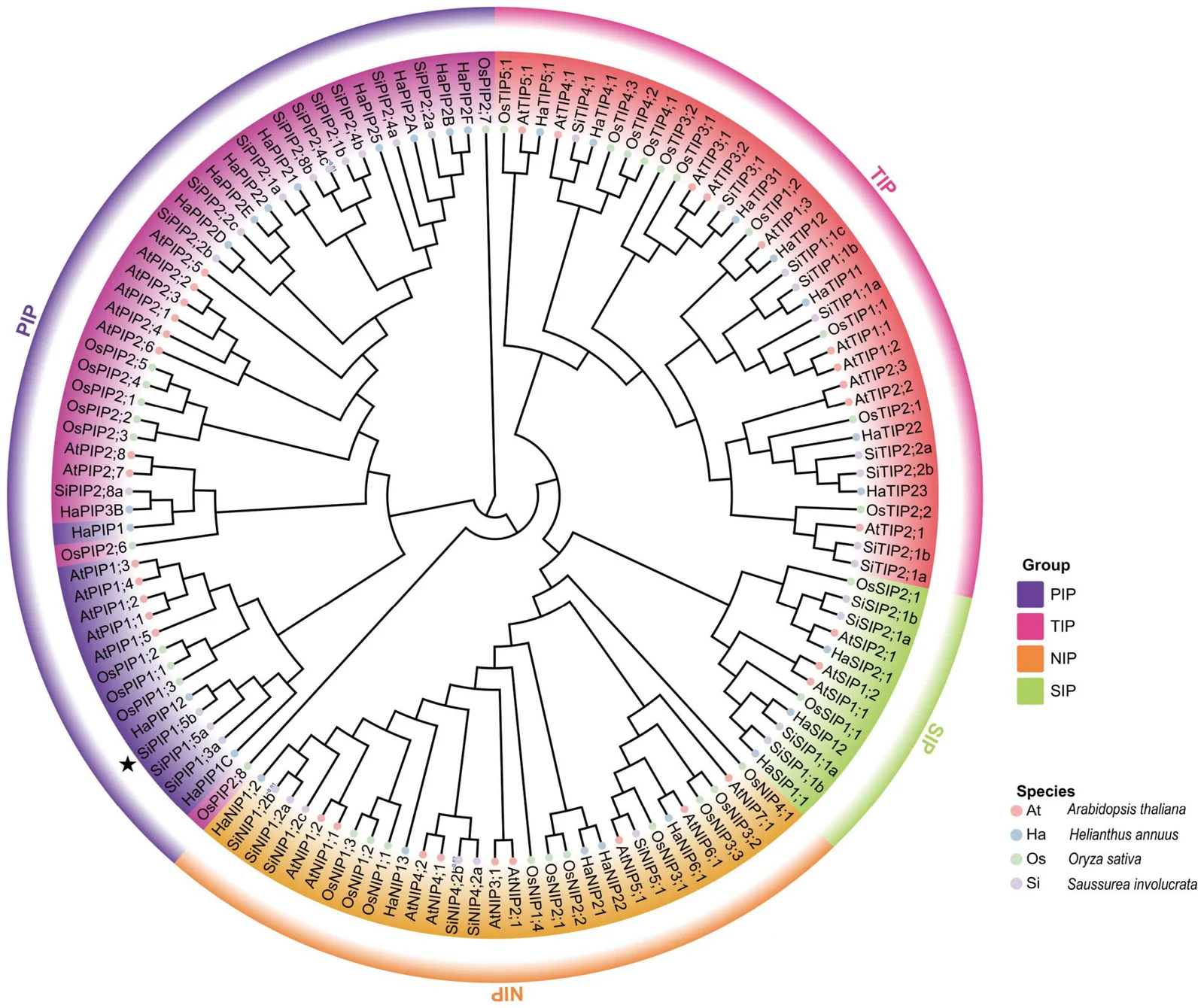
Phylogenetic analysis of aquaporin proteins from four plant species. Neighbor-joining (NJ) phylogenetic tree of aquaporin proteins from *Arabidopsis thaliana* (At), *Helianthus annuus* (Ha), *Oryza sativa* (Os), and *Saussurea involucrata* (Si). Aquaporins are classified into four subfamilies, including plasma membrane intrinsic proteins (PIPs), tonoplast intrinsic proteins (TIPs), nodulin26-like intrinsic proteins (NIPs), and small basic intrinsic proteins (SIPs), which are highlighted by different colors. *SiPIP1;5b* is indicated by a black star. The scale bar represents evolutionary distance.

To determine the evolutionary position of *SiPIP1;5b*, aquaporin proteins from *Saussurea involucrata*, *Arabidopsis thaliana*, *Helianthus annuus*, and *Oryza sativa* were subjected to phylogenetic analysis. The resulting phylogenetic tree clearly separated the aquaporins into four major subfamilies, namely plasma membrane intrinsic proteins (PIPs), tonoplast intrinsic proteins (TIPs), nodulin 26-like intrinsic proteins (NIPs), and small basic intrinsic proteins (SIPs) (Figure 1). *SiPIP1;5b* clustered within the PIP1 subgroup and showed a relatively close phylogenetic relationship with PIP1 homologs from sunflower, consistent with the close taxonomic relationship between *S. involucrata* and *H. annuus*.

**Figure 2:**
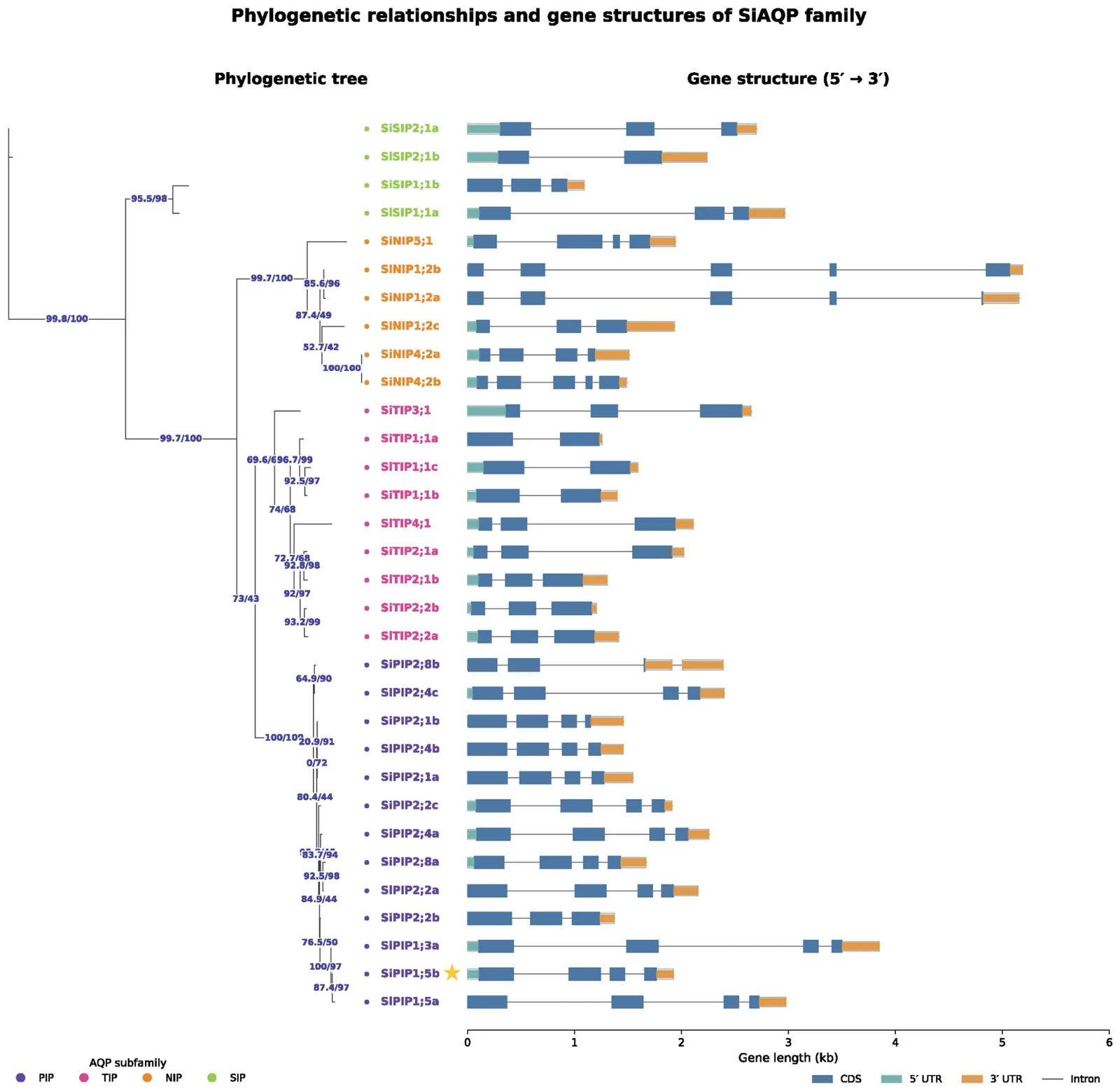
Phylogenetic relationships and gene structures of the SiAQP family. Phylogenetic tree and exon–intron organization of SiAQP genes. Exons are shown as blue boxes, untranslated regions (UTRs) as orange boxes, and introns as black lines. Gene structures are displayed from the 5′ to 3′ direction. *SiPIP1;5b* is highlighted in yellow and marked with a yellow star.

Gene structure analysis of the *SiAQP* family further showed that genes located within the same phylogenetic branch generally exhibited similar exon–intron organizations (Figure 2). *SiPIP1;5b* displayed the characteristic gene structure of the SiPIP1 subgroup, with an exon–intron arrangement similar to those of closely related PIP1 genes, further supporting its classification as a PIP1-type.

**Figure 3:**
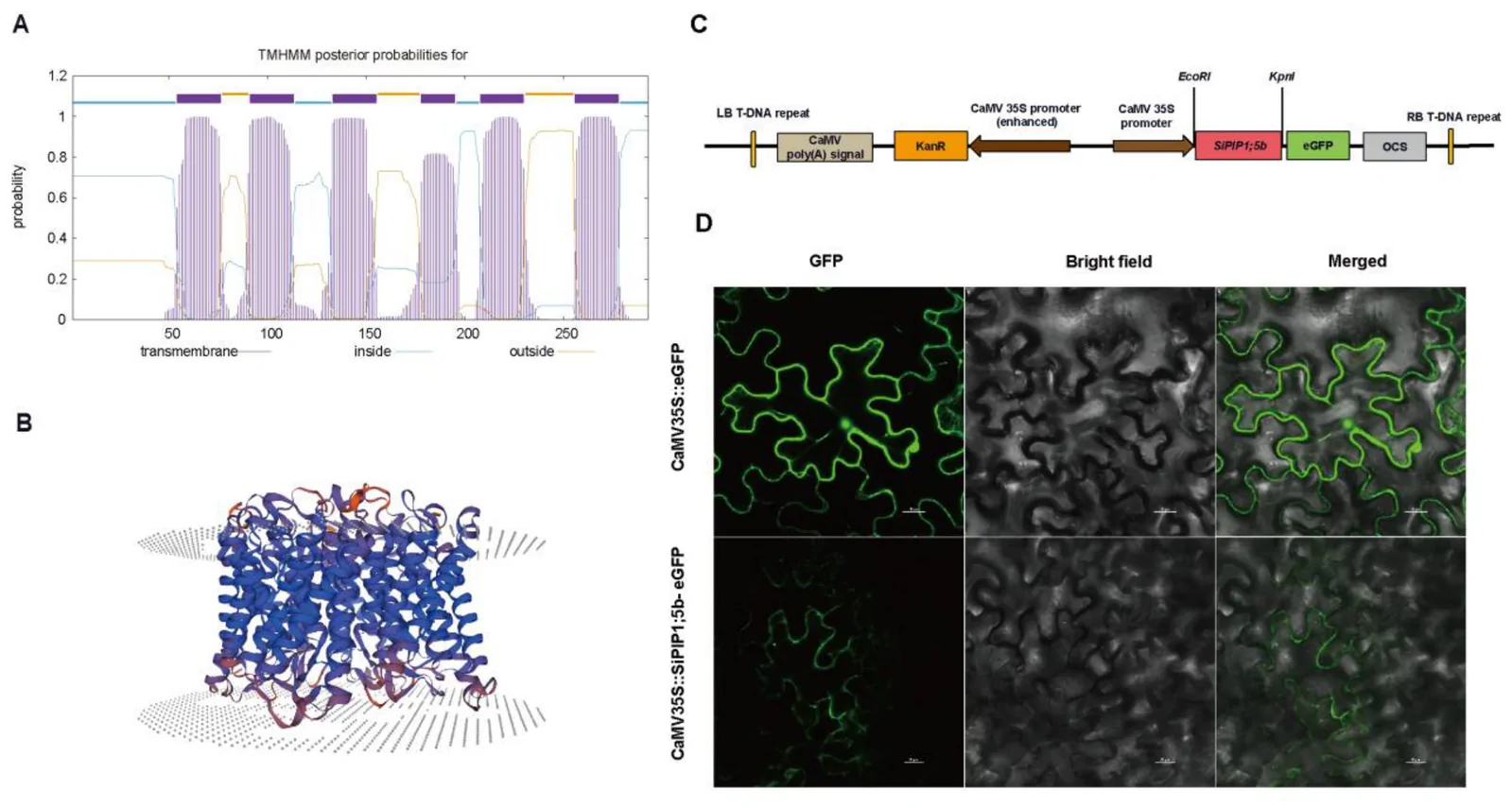
Structural features, expression constructs, and subcellular localization of *SiPIP1;5b*. (**A**) Predicted transmembrane topology of *SiPIP1;5b* generated using the TMHMM. (**B**) Predicted three-dimensional structure of *SiPIP1;5b*. (**C**) Schematic representation of the pCaMV35S::SiPIP1;5b-eGFP construct. (**D**) Transient expression of free eGFP and SiPIP1;5b-eGFP in *Nicotiana benthamiana* leaf epidermal cells. GFP fluorescence, bright-field, and merged images are shown in the figure. Scale bars = 20 μm.

Prediction of membrane topology showed that *SiPIP1;5b* contains six typical transmembrane α-helices, with both the N- and C-terminal regions predicted to face the cytoplasmic side of the membrane (Figure 3A). Three-dimensional structural modeling revealed a typical aquaporin-like fold consisting of six membrane-spanning helices and two short half-helices arranged around a central channel region (Figure 3B). These structural features are characteristic of members of the major intrinsic protein superfamily.

To examine the subcellular distribution of *SiPIP1;5b*, the *SiPIP1;5b* coding sequence was fused to eGFP and transiently expressed in *Nicotiana benthamiana* leaf epidermal cells. Free eGFP fluorescence was broadly distributed in the cytoplasm and nucleus, whereas SiPIP1;5b-eGFP fluorescence was predominantly observed along the cell periphery, outlining the cellular boundary (Figure 3C,D). This peripheral fluorescence pattern is consistent with plasma-membrane localization of *SiPIP1;5b*, although definitive confirmation of plasma-membrane targeting would require co-localization with a plasma-membrane marker or plasmolysis analysis.

### 2.2. Generation and Selection of SiPIP1;5b-Overexpressing Arabidopsis Lines

Six independent *SiPIP1;5b*-overexpressing *Arabidopsis* lines were obtained through Agrobacterium-mediated transformation, antibiotic selection, and molecular verification (Figure S1). In total, 14 primary T1 transformants were initially screened by PCR, and six positive independent lines were retained for subsequent qRT-PCR expression analysis. *SiPIP1;5b* transcripts were not detected in wild-type Col-0 plants, whereas all transgenic lines showed detectable expression levels.

Transcript abundance varied among the independent lines, with OE-2 showing the highest expression, followed by OE-1 and OE-3. Based on their relatively high and stable expression levels, OE-1, OE-2, and OE-3 were selected for subsequent freezing-tolerance analyses (Figure S1).

### 2.3. Overexpression of SiPIP1;5b Enhances Freezing Tolerance in Arabidopsis

**Figure 4:**
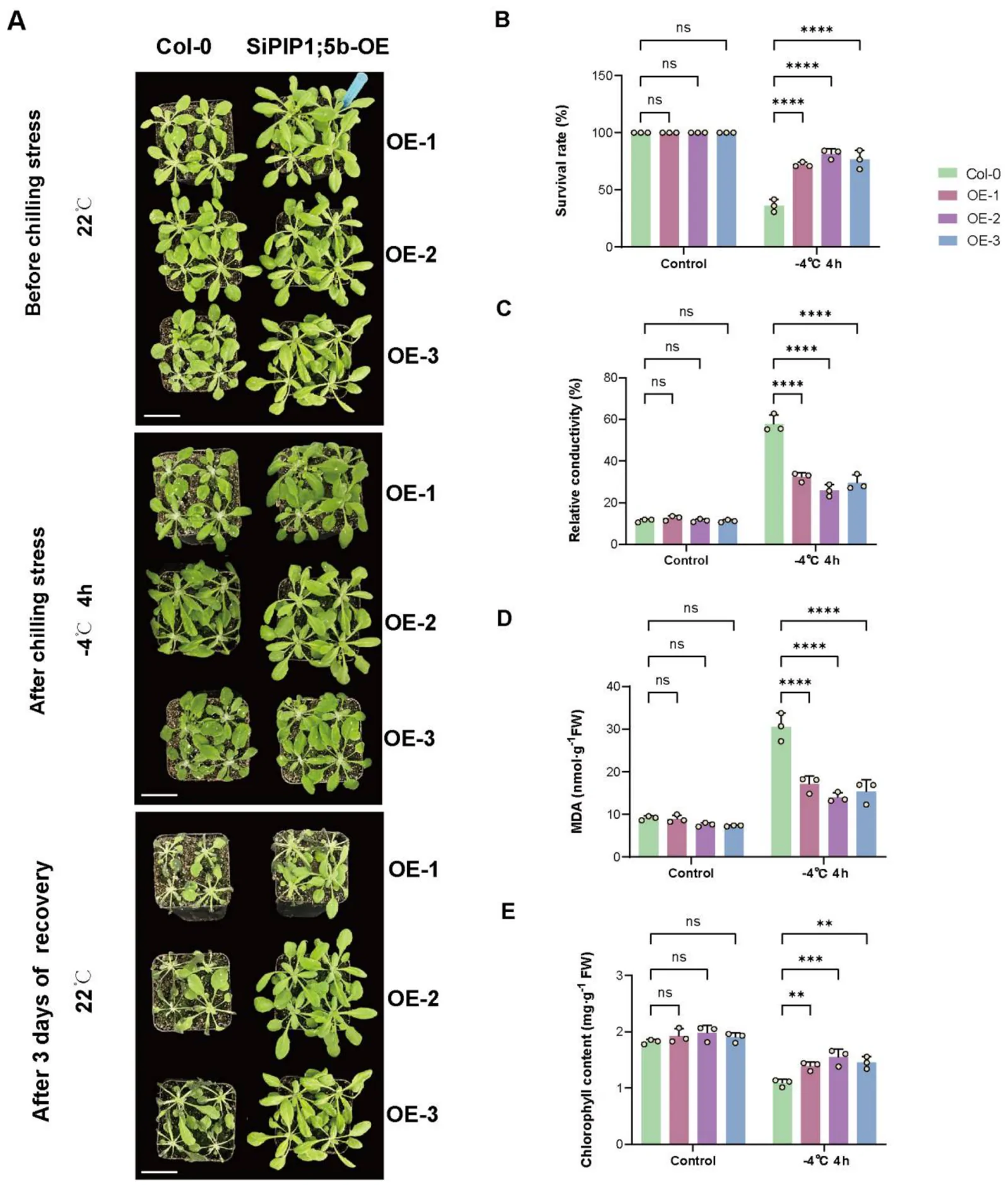
Overexpression of *SiPIP1;5b* enhances freezing tolerance in *Arabidopsis*. (**A**) Phenotypes of four-week-old wild-type Col-0 and three independent *SiPIP1;5b*-overexpressing lines (OE-1, OE-2, and OE-3) under control conditions (22 °C), immediately after freezing treatment at −4 °C for 4 h, and after 3 d of recovery at 22 °C. Scale bars = 4 cm. (**B**) Survival rate after recovery. (**C**) Relative electrolyte conductivity. (**D**) Malondialdehyde (MDA) content. (**E**) Chlorophyll content. Physiological measurements were performed immediately after freezing treatment. For the survival-rate assay, 12 individual plants were assessed per biological replicate for each genotype. Data are presented as means ± SEM from three independent biological replicates. Statistical significance was assessed by two-way ANOVA followed by Dunnett’s multiple-comparison test. ns, not significant; ** *p* < 0.01; *** *p* < 0.001; **** *p* < 0.0001.

Under normal conditions, no obvious phenotypic differences were observed between Col-0 and the three *SiPIP1;5b*-overexpressing lines. After exposure to −4 °C for 4 h, Col-0 plants displayed severe wilting and water-soaked leaf damage, whereas the OE lines showed substantially milder injury. After 3 d of recovery, most transgenic plants resumed growth, while the survival of Col-0 was markedly lower (Figure 4A,B). The survival rate increased from approximately 36% in Col-0 to 72–82% in the transgenic lines. For survival-rate determination, 12 individual plants per genotype were scored in each of the three independent biological replicates.

Freezing caused a pronounced increase in relative electrolyte leakage and MDA content in Col-0, indicating substantial membrane injury and lipid peroxidation. Both parameters were markedly lower in the three OE lines following freezing treatment (Figure 4C,D). In contrast, chlorophyll content was better maintained in the transgenic plants than in Col-0 after stress (Figure 4E).

Together, these results indicate that *SiPIP1;5b* overexpression enhances freezing survival and is associated with reduced freezing-induced cellular and membrane damage in *Arabidopsis* plants.

### 2.4. SiPIP1;5b Overexpression Is Associated with Reduced ROS Accumulation and Enhanced Stress-Related Defense Responses Under Freezing Stress

**Figure 5:**
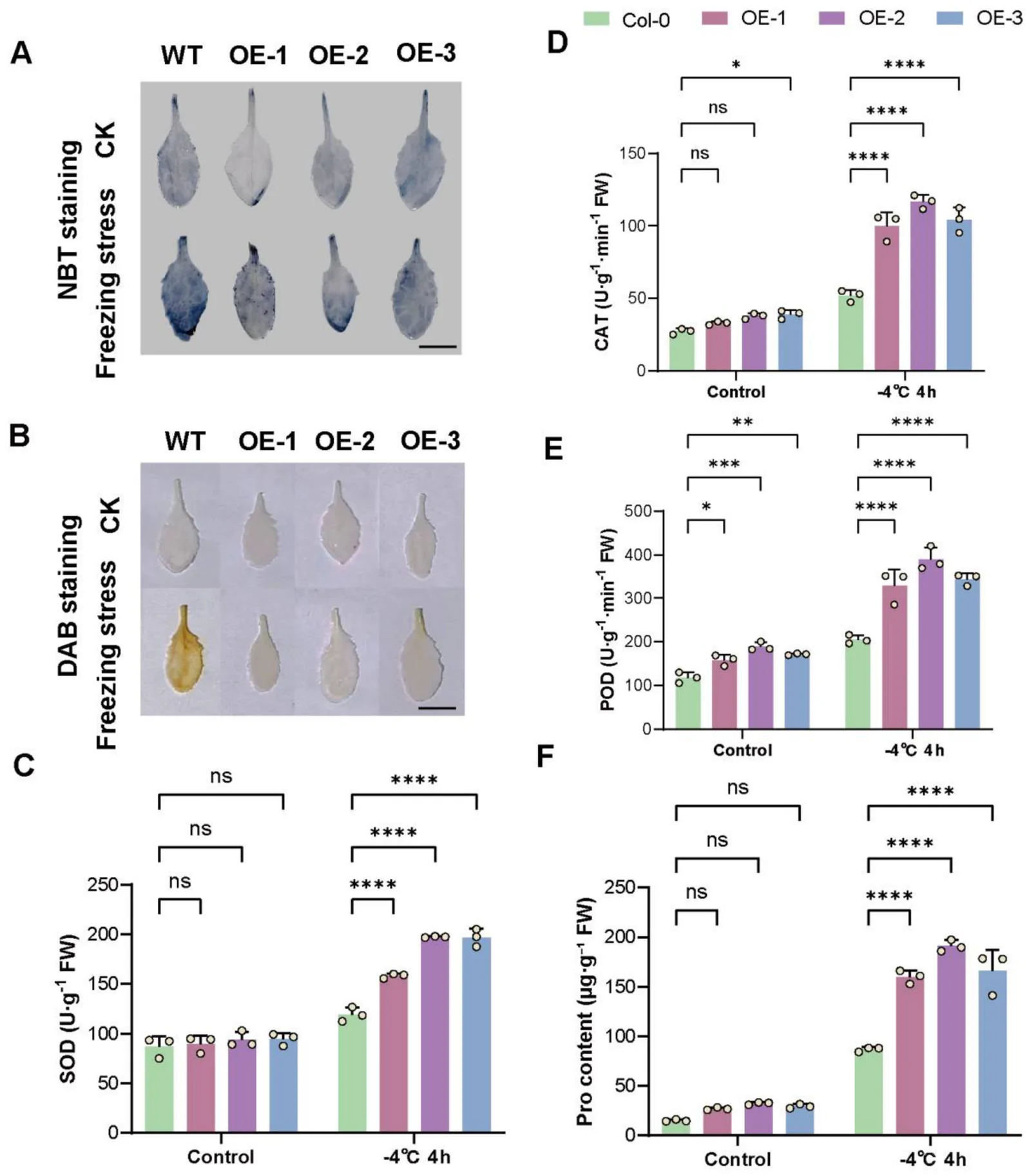
Effects of *SiPIP1;5b* overexpression on ROS accumulation, antioxidant enzyme activities, and proline content in *Arabidopsis* under freezing stress. (**A**,**B**) Histochemical detection of superoxide (O_2_•^−^) by nitroblue tetrazolium (NBT) staining (**A**) and hydrogen peroxide (H_2_O_2_) by 3,3′-diaminobenzidine (DAB) staining (**B**) in leaves of Col-0 and *SiPIP1;5b*-overexpressing lines under control conditions or after freezing treatment at −4 °C for 4 h. Scale bars = 1 cm. The staining results represent qualitative/semi-quantitative evidence of ROS accumulation. (**C**–**F**) SOD activity (**C**), CAT activity (**D**), POD activity (**E**), and proline content (**F**). Data are presented as means ± SEM from three independent biological replicates. Statistical significance was assessed by two-way ANOVA followed by Dunnett’s multiple-comparison test. ns, not significant; * *p* < 0.05; ** *p* < 0.01; *** *p* < 0.001; **** *p* < 0.0001.

NBT and DAB staining showed little ROS accumulation under control conditions in either Col-0 or the transgenic lines. Following freezing treatment, Col-0 leaves exhibited much stronger blue and brown staining than the OE lines, indicating greater accumulation of O_2_•^−^ and H_2_O_2_, respectively (Figure 5A,B). These histochemical stainings provide qualitative/semi-quantitative evidence of ROS accumulation.

Freezing also increased SOD, CAT, and POD activities in all genotypes, but the increases were generally greater in the *SiPIP1;5b*-overexpressing lines. Proline accumulation was likewise substantially higher in the transgenic plants, particularly after freezing treatment (Figure 5C–F).

These results indicate that improved freezing tolerance in *SiPIP1;5b*-overexpressing *Arabidopsis* is associated with reduced ROS accumulation, enhanced antioxidant responses, and increased osmotic adjustment.

### 2.5. Generation and Selection of SiPIP1;5b-Overexpressing Tomato Lines

Seven independent *SiPIP1;5b*-overexpressing tomato lines were obtained by *Agrobacterium*-mediated transformation. No detectable *SiPIP1;5b* transcripts were observed in wild-type Yaxin87-5 plants, whereas expression varied substantially among the transgenic lines.

OE-2 showed the highest transcript abundance, followed by OE-1 and OE-3. These three lines exhibited relatively high and stable expression and were therefore selected for subsequent chilling tolerance analyses (Figure S2).

### 2.6. Overexpression of SiPIP1;5b Enhances Chilling Tolerance in Tomato

**Figure 6:**
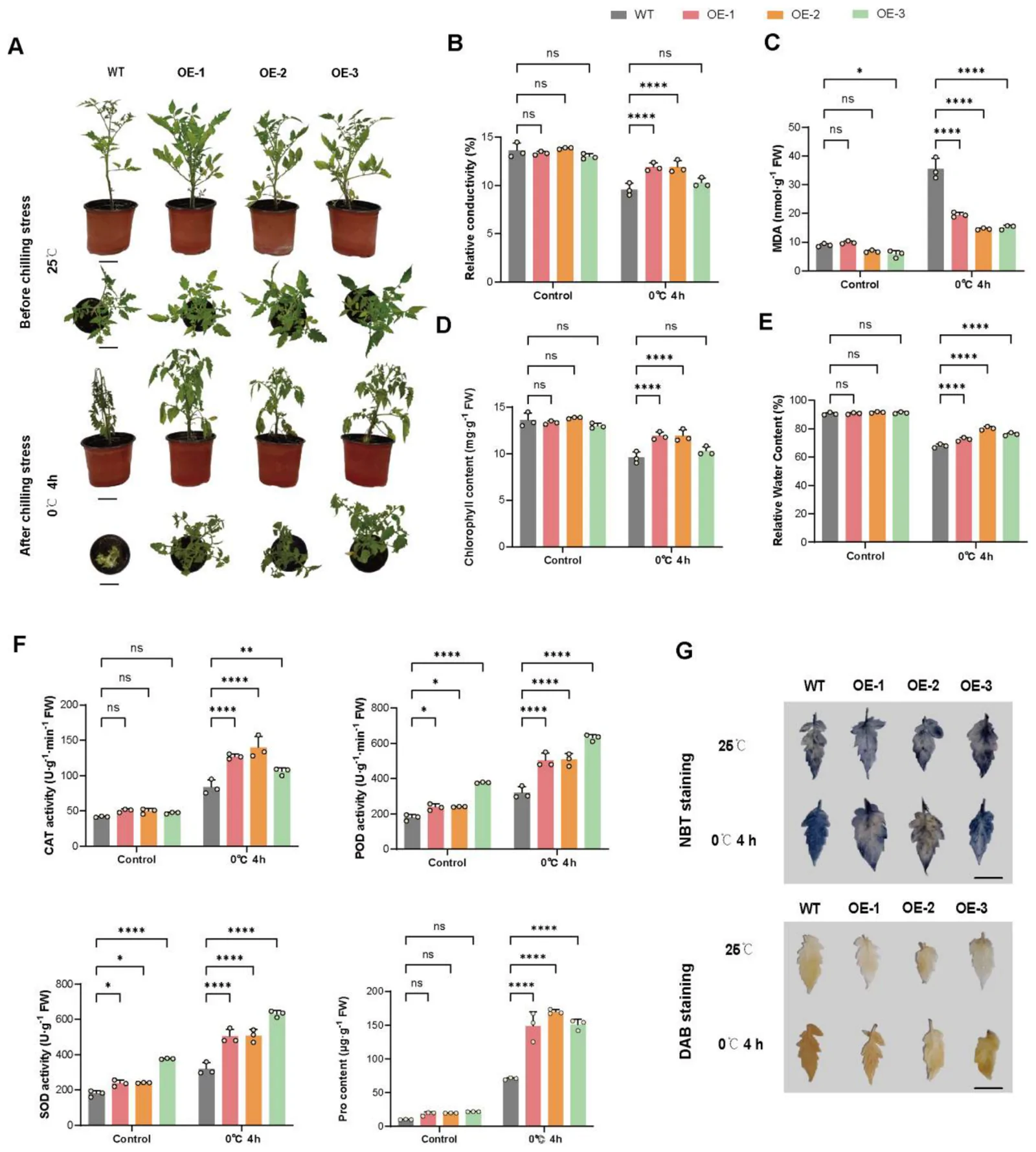
Overexpression of *SiPIP1;5b* enhances chilling tolerance in tomato. (**A**) Phenotypes of five-week-old wild-type (WT) and three independent *SiPIP1;5b*-overexpressing lines (OE-1, OE-2, and OE-3) before and after chilling treatment at 0 °C for 4 h. Scale bars = 7 cm. (**B**–**E**) Relative electrolyte conductivity (**B**), MDA content (**C**), chlorophyll content (**D**), and relative water content (**E**). (**F**) CAT, POD, and SOD activities and proline content. (**G**) NBT and DAB staining for O_2_•^−^ and H_2_O_2_ accumulation, respectively. Scale bars = 2 cm. The staining results represent qualitative/semi-quantitative evidence of ROS accumulation. Data are presented as means ± SEM from three independent biological replicates. Statistical significance was assessed by two-way ANOVA followed by Dunnett’s multiple-comparison test using WT as the reference genotype. ns, not significant; * *p* < 0.05; ** *p* < 0.01; **** *p* < 0.0001.

Under normal conditions, wild-type and transgenic tomato plants exhibited similar overall morphology. Following exposure to 0 °C for 4 h, wild-type plants exhibited pronounced leaf wilting, drooping, and water-soaked injury, whereas the three *SiPIP1;5b*-overexpressing lines showed considerably milder symptoms (Figure 6A).

Chilling caused marked increases in relative electrolyte leakage and MDA content in wild-type plants, whereas both parameters were substantially lower in the transgenic lines (Figure 6B,C). Chlorophyll content and relative water content were generally better maintained in OE plants, although the magnitude of the response differed among independent lines (Figure 6D,E).

These results indicate that *SiPIP1;5b* overexpression enhances chilling tolerance in tomato and is associated with reduced cellular injury and improved maintenance of tissue water status.

### 2.7. SiPIP1;5b Overexpression Modifies Antioxidant Responses and Reduces ROS Accumulation in Tomato Under Chilling Stress

Following chilling treatment, CAT and POD activities were generally higher in transgenic tomato lines than in the wild type, whereas SOD activity did not show a consistent genotype-dependent increase. Proline accumulation was strongly enhanced in all three OE lines after chilling (Figure 6F).

Consistent with these biochemical responses, NBT and DAB staining showed substantially weaker accumulation of O_2_•^−^ and H_2_O_2_ in transgenic leaves than in wild-type leaves after chilling treatment (Figure 6G).

Thus, the enhanced chilling tolerance of *SiPIP1;5b*-overexpressing tomato plants is associated with reduced ROS accumulation and strengthened stress-related antioxidant and osmotic responses. The lack of a consistent increase in SOD activity also suggests that the protective effect is not simply attributable to the uniform activation of all antioxidant enzymes.

### 2.8. SiPIP1;5b Overexpression Improves Agronomic Performance of Tomato Under Field Conditions

**Figure 7:**
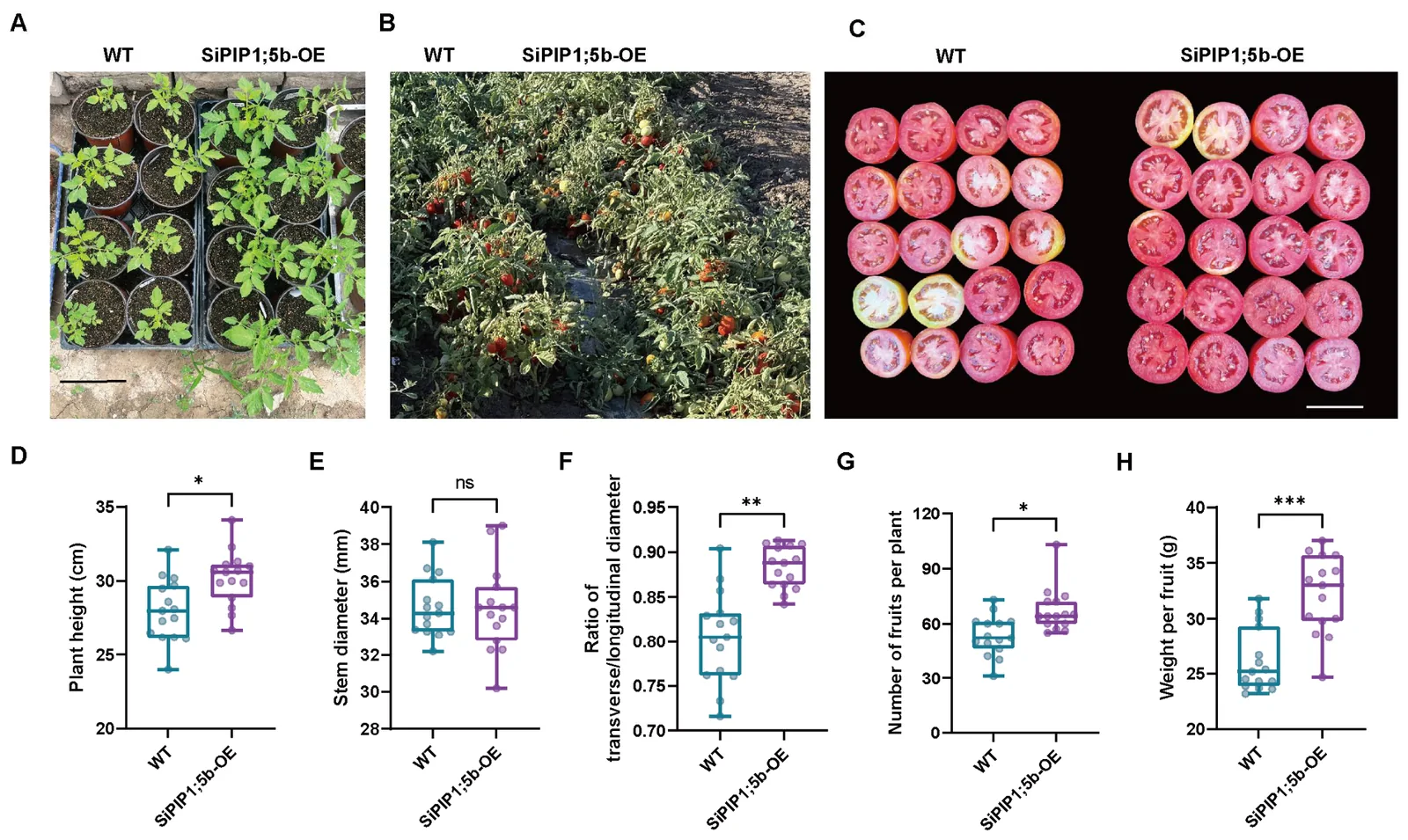
Field performance and agronomic traits of T3-generation *SiPIP1;5b*-overexpressing tomato plants. (**A**) Representative phenotypes of wild-type (WT) and *SiPIP1;5b*-overexpressing tomato plants. Scale bar = 15 cm. (**B**) Representative plants grown under field conditions in 2024. (**C**) Cross-sections of mature fruits. Scale bar = 5 cm. (**D**–**H**) Plant height (**D**), stem diameter (**E**), fruit shape index, calculated as transverse diameter/longitudinal diameter (**F**), fruit number per plant (**G**), and individual fruit weight (**H**). Fifteen plants were evaluated per genotype (*n* = 15; five plants per plot in each of the three replicate blocks). For fruit shape index and individual fruit weight, six mature representative fruits were harvested per plant, and the mean value per plant was used in the statistical analysis. Data are shown as box plots. Because the plot is the experimental unit of the randomized complete block design, statistical significance refers to the comparison of the three plot means per genotype (five plants per plot; two-tailed Student’s *t*-test): * *p* < 0.05; ** *p* < 0.01; *** *p* < 0.001; ns, not significant. The corresponding two-way ANOVA of the plant-level data, with genotype and block as factors, is given in Table S3; the block effect was not significant for any trait (*p* > 0.05).

T3-generation *SiPIP1;5b*-overexpressing tomato plants and wild-type Yaxin87-5 plants were evaluated in a single field trial conducted in 2024 at Shihezi, Xinjiang, China. The transgenic plants exhibited more vigorous growth and greater plant height than wild type, whereas stem diameter was not significantly affected (Figure 7A,B,D,E).

At maturity, the fruits from the transgenic plants were more rounded, as reflected by a significantly higher fruit shape index (Figure 7C,F). In addition, *SiPIP1;5b*-overexpressing plants produced more fruits per plant and showed greater individual fruit weights than wild-type plants (Figure 7G,H).

These results indicate that constitutive *SiPIP1;5b* expression did not impose an obvious growth penalty under our tested 2024 Shihezi field conditions and was associated with improved vegetative and reproductive performance in that environment. Because these data were obtained at a single location and in a single growing season, the results should not be generalized beyond these tested conditions.

## 3. Discussion

### 3.1. SiPIP1;5b Is a PIP1 Aquaporin Associated with Low-Temperature Tolerance

Aquaporins are important regulators of membrane water permeability and enable plants to rapidly adjust cellular and whole-plant water relations in response to environmental changes [7,31]. Among plant aquaporins, plasma membrane intrinsic proteins (PIPs) constitute a major subfamily and are regulated at multiple levels, including transcription, phosphorylation, gating, heteromerization, and membrane trafficking [9]. In the present study, *SiPIP1;5b,* clustered within the PIP1 subgroup, contained the characteristic six-transmembrane architecture of major intrinsic proteins and showed a fluorescence distribution consistent with plasma-membrane localization. Together, these features support its classification as a PIP1-type aquaporin.

Overexpression of *SiPIP1;5b* enhanced freezing tolerance in *Arabidopsis* and chilling tolerance in tomato, suggesting that the protective effect of this PIP1-type aquaporin is conserved across different plant species. This observation is consistent with previous studies showing that individual PIP isoforms participate in low-temperature responses. In *Arabidopsis*, *PIP1;4* and *PIP2;5* contribute to cold acclimation, root growth, and survival after freezing, suggesting coordinated but isoform-specific functions of PIPs during cold adaptation [17].

Previous analysis of the *S. involucrata* AQP family revealed distinct expression patterns between the two closely related *PIP1;5* genes. *SiPIP1;5a* was induced under both chilling and freezing conditions, whereas *SiPIP1;5b* mainly responded to chilling stress. Moreover, *SiPIP1;5a* overexpression enhanced low-temperature tolerance in tomato [29].

In the present study, *SiPIP1;5b* conferred freezing tolerance to *Arabidopsis* and chilling tolerance to tomato. These results suggest that closely related Si*PIP1;5* isoforms may share conserved protective functions, while being differentially regulated in *S. involucrata*.

Nevertheless, the effects of PIPs under freezing conditions are isoform-dependent. For example, overexpression of *Rhododendron catawbiense* PIP2 genes reduced freezing tolerance in *Arabidopsis* [32]. Such contrasting observations indicate that PIP function during low-temperature stress depends on the properties of individual isoforms and their physiological context rather than simply on total aquaporin abundance.

### 3.2. SiPIP1;5b May Enhance Cold Tolerance by Maintaining Membrane Integrity and Water Status

Low temperature strongly affects cellular water balance and plasma membrane stability. During freezing, extracellular ice formation promotes cellular dehydration, whereas chilling alters membrane properties and restricts normal water transport. Therefore, maintenance of membrane integrity and appropriate water redistribution are important components of low-temperature tolerance.

In the present study, *SiPIP1;5b*-overexpressing *Arabidopsis* plants showed substantially lower relative electrolyte leakage and MDA accumulation after freezing, along with higher survival and better chlorophyll retention. Similarly, transgenic tomato plants showed reduced electrolyte conductivity and MDA accumulation following chilling, whereas several OE lines maintained a higher relative water content. These physiological responses are consistent with reduced membrane injury and improved maintenance of the cellular water status in these plants.

Because PIPs regulate transmembrane water movement, *SiPIP1;5b* may contribute to more efficient water redistribution during low-temperature stress and recovery. The regulation of plant hydraulic conductivity by aquaporins is well established and represents an important mechanism by which plants adjust to environmental changes [8,31].

### 3.3. SiPIP1;5b Is Associated with Improved ROS Homeostasis Under Low-Temperature Stress

Excessive accumulation of reactive oxygen species is a major consequence of low-temperature stress and can promote lipid peroxidation, membrane damage, and loss of cellular function. In both *Arabidopsis* and tomato, *SiPIP1;5b* overexpression was accompanied by weaker NBT and DAB staining under low-temperature stress, indicating reduced accumulation of O_2_•^−^ and H_2_O_2_. This response was accompanied by enhanced activities of several antioxidant enzymes and increased proline accumulation.

Antioxidant responses differed somewhat between the two species. In *Arabidopsis*, SOD, CAT, and POD activities were generally higher in the OE lines following freezing, whereas in tomato, CAT and POD showed more consistent increases than SOD. This difference suggests that *SiPIP1;5b* does not simply activate a uniform antioxidant program. Instead, the protective effect may involve an upstream physiological process that modifies ROS metabolism in a species- and stress-dependent manner.

An additional possibility is that *SiPIP1;5b* participates directly in redox signaling. Several PIP aquaporins are permeable to both water and H_2_O_2_, allowing them to contribute to transmembrane redox signaling [33,34,35]. *Arabidopsis* PIP1 and PIP2 members have also been shown to facilitate physiologically relevant H_2_O_2_ diffusion in roots [36]. More recent mechanistic studies further support the ability of PIP channels to transport both water and H_2_O_2_ through related but distinct permeation mechanisms [37].

Previous studies on *S. involucrata* have also proposed a relationship among PIP1 proteins, PIP1/PIP2 complex formation, and ROS signaling [29].

Based on the present results, *SiPIP1;5b* may contribute to the coordination of hydraulic and redox responses under low-temperature stress. Whether *SiPIP1;5b* transports H_2_O_2_ remains an interesting question for further functional analysis. Taken together, the present data establish an association between *SiPIP1;5b* expression and improved low-temperature tolerance rather than a defined molecular mechanism; direct measurements of water and H_2_O_2_ permeability, as well as characterisation of the PIP1/PIP2 complexes containing *SiPIP1;5b*, will be required to demonstrate the mechanism.

### 3.4. SiPIP1;5b Combines Low-Temperature Tolerance with Favorable Agronomic Performance in Tomato

In addition to improving chilling tolerance, *SiPIP1;5b* overexpression was associated with favorable agronomic traits under field conditions. T3 transgenic tomato plants showed increased plant height, fruit number per plant, and individual fruit weight, whereas stem diameter was not significantly affected. These results indicate that constitutive expression of *SiPIP1;5b* did not impose an obvious growth penalty under the tested field conditions at a single location and in a single growing season (Shihezi, 2024).

This finding is particularly interesting when compared with previous results for closely related *SiPIP1;5a*. Overexpression of *SiPIP1;5a* improved tomato tolerance to low temperatures but was accompanied by a dwarf phenotype under normal growth conditions [29].

In contrast, *SiPIP1;5b*-overexpressing plants maintained normal growth and showed improved reproductive performance compared to WT plants. This difference suggests that closely related *PIP1;5* isoforms may differ in their effects on the growth–stress balance, possibly through differences in transport properties, regulation, or interactions with endogenous PIPs.

Aquaporins are increasingly regarded as integrators of transport, signaling, and stress acclimation, rather than solely as passive water channels. Recent studies emphasize the involvement of PIPs in water and solute transport, redox signaling, post-translational regulation, and abiotic stress adaptation [38]. Therefore, the combined stress tolerance and field phenotypes observed here suggest that *SiPIP1;5b* is a promising candidate for further investigation of aquaporin-mediated coordination between stress resilience and plant productivity.

## 4. Materials and Methods

### 4.1. Plant Materials and Growth Conditions

*Arabidopsis thaliana* ecotype Columbia-0 (Col-0) was used as the wild type for genetic transformation and functional analyses. Tomato (*Solanum lycopersicum* L. cv. Yaxin87-5) seeds maintained in our laboratory were used for heterologous expression, physiological analyses, and field evaluation. *Saussurea involucrata* plant material maintained in our laboratory was used as the source of the *SiPIP1;5b* gene. *Nicotiana benthamiana* plants were used for transient expression assays.

*Arabidopsis* seeds were surface-sterilized and germinated on half-strength Murashige and Skoog (1/2 MS) medium supplemented with 1% (*w*/*v*) sucrose and 0.8% (*w*/*v*) agar. After stratification at 4 °C for 3 d, seedlings were grown at 22 ± 1 °C under a 16 h light/8 h dark photoperiod, with a photon flux density of 120 μmol m^−2^ s^−1^ and approximately 60% relative humidity. Ten-day-old seedlings were transplanted into 7 × 7 cm pots containing a 1:1 (*v*/*v*) mixture of nutrient soil and vermiculite and grown for four weeks before freezing treatment.

Tomato (*Solanum lycopersicum* L. cv. Yaxin87-5) seeds were surface-sterilized and germinated in plug trays filled with a commercial peat-based substrate in a growth room at 25 °C under a 16 h light/8 h dark photoperiod provided by fluorescent lamps (light intensity of approximately 70 µmol m^−2^ s^−1^) and 60–70% relative humidity. At the four-leaf stage, seedlings were transplanted into plastic pots (20 cm in diameter and 20 cm in height) filled with a sterilized mixture of peat, vermiculite and soil (1:1:1, *v*/*v*/*v*) and grown in a naturally lit greenhouse at 25/22 °C (day/night) under a 16 h light/8 h dark photoperiod; daylight was supplemented with fluorescent lamps to maintain a minimum photon flux density of approximately 300 µmol m^−2^ s^−1^ at plant height, and relative humidity was maintained at 60–70%. Plants were irrigated with approximately 1 L of tap water per pot every 3 d and fertilized once a week with half-strength Hoagland nutrient solution. No cold-acclimation or other low-temperature pretreatment was applied: plants were grown for five weeks under these conditions and then directly exposed to the chilling treatment.

### 4.2. Cloning and Bioinformatic Analysis of SiPIP1;5b

The full-length coding sequence (CDS) of *SiPIP1;5b* was amplified from *S. involucrata* leaf cDNA using gene-specific primers designed based on transcriptome sequencing data. The amplified fragment was cloned into the pMD19-T vector and verified by Sanger sequencing.

Transmembrane helices of *SiPIP1;5b* were predicted using TMHMM Server v2.0. The three-dimensional structure was predicted with AlphaFold2 (ColabFold v1.5.2, default parameters); the model-confidence (pLDDT) score of the predicted *SiPIP1;5b* structure was mostly > 80, indicating high-confidence modeling, and the model was visualized using PyMOL. Aquaporin protein sequences were retrieved from UniProtKB (reviewed/Swiss-Prot entries for *A. thaliana* and *O. sativa*, and reviewed and unreviewed Swiss-Prot/TrEMBL entries for *H. annuus*; UniProtKB release 2026_03), whereas the sequences of *S. involucrata* were taken from our in-house transcriptome and genome assembly dataset; the accession numbers of all aquaporin sequences used for phylogenetic analysis are listed in Table S2. Multiple sequence alignment was performed using ClustalW, and a neighbor-joining phylogenetic tree was constructed using MEGA X with 1000 bootstrap replicates. Gene structures were analyzed using GSDS 2.0 and visualized using TBtools; the exon–intron organization of the SiAQP genes was determined from the genomic sequences and gene identifiers of the *S. involucrata* in-house genome assembly.

### 4.3. Vector Construction and Plant Transformation

The verified *SiPIP1;5b* coding sequence was inserted into the plant expression vector pCAMBIA2300-35S-eGFP through EcoRI and KpnI restriction sites to generate pCaMV35S::SiPIP1;5b-eGFP, which was specifically used for subcellular localization analysis via transient expression in tobacco leaves. For stable genetic transformation of *Arabidopsis* and tomato, the confirmed *SiPIP1;5b* coding sequence was inserted into the pCAMBIA1300-35S vector to construct the pCaMV35S::*SiPIP1;5b* overexpression vector. All recombinant constructs were confirmed by restriction digestion and DNA sequencing and were introduced into *Agrobacterium tumefaciens* strain GV3101 using the freeze–thaw method.

*Arabidopsis* transformation was performed using the floral dip method. Flowering plants were immersed for 30 s in an *Agrobacterium* suspension (OD_600_ = 0.8) containing 5% (*w*/*v*) sucrose and 0.05% (*v*/*v*) Silwet L-77. T0 seeds were harvested after maturation and screened on 1/2 MS medium containing 50 mg L^−1^ kanamycin. Positive transformants were confirmed by PCR and qRT-PCR. Three independent homozygous T3 lines, OE-1, OE-2, and OE-3, were used for subsequent experiments.

Tomato transformation was performed using an *Agrobacterium*-mediated hypocotyl transformation method. Hypocotyl explants were excised from 8-day-old seedlings of cv. Yaxin87-5 and cultured on shoot-induction medium consisting of MS medium supplemented with 2.0 mg L^−1^ 6-benzylaminopurine (6-BA), 0.15 mg L^−1^ indole-3-acetic acid (IAA), 50 mg L^−1^ kanamycin, and 200 mg L^−1^ timentin. Regenerated shoots were transferred to half-strength MS rooting medium supplemented with 0.3 mg L^−1^ IAA, 50 mg L^−1^ kanamycin, and 200 mg L^−1^ timentin. Independent transgenic plants were verified by PCR and qRT-PCR before further analyses.

### 4.4. RNA Extraction and Quantitative Real-Time PCR Analysis

Total RNA was extracted from young leaves of *Arabidopsis* and tomato using TransZol Up reagent (TransGen Biotech, Beijing, China; Cat. No. ET111) according to the manufacturer’s instructions. RNA concentration and purity were assessed using a NanoDrop spectrophotometer (Thermo Fisher Scientific, Waltham, MA, USA), and RNA integrity was evaluated using 1% agarose gel electrophoresis.

First-strand cDNA was synthesized from 1 μg of total RNA using TransScript^®^ All-in-One First-Strand cDNA Synthesis SuperMix for qPCR (One-Step gDNA Removal) (TransGen Biotech, Beijing, China; Cat. No. AT341) according to the manufacturer’s protocol.

Quantitative real-time PCR (qRT-PCR) was performed using a Roche LightCycler^®^ 480 system (Roche, Salt Lake City, UT, USA) with SYBR Green Real-Time PCR Master Mix (KAPA Biosystems, Wilmington, MA, USA). Each 20-μL reaction contained 2 μL of diluted cDNA, 0.4 μL of each primer (10 μM), 10 μL of 2× SYBR Green Master Mix, and 7.2 μL of nuclease-free water. The amplification program consisted of an initial denaturation at 95 °C for 3 min, followed by 40 cycles of 95 °C for 10 s, 60 °C for 20 s, and 72 °C for 20 s. A melting-curve analysis from 65 °C to 95 °C was performed to verify amplification specificity.

*AtACT2* was used as the internal reference gene for *Arabidopsis* and *SlACT* for tomato qRT-PCR analyses; the *AtACT7* primer pair was retained as an alternative reference gene and was not used in the present study. The suitability of the reference genes was verified in the present experiment: melting-curve analysis showed a single amplification product for each primer pair, and the Ct values of *AtACT2* and *SlACT* were stable across all genotypes and temperature treatments, with no genotype- or treatment-dependent variation. In addition, both genes have been validated as reliable reference genes for transcript normalisation in *Arabidopsis* and tomato [39,40]. Three independent biological replicates were analyzed for each genotype, with three technical replicates per biological sample. Relative transcript abundance was calculated using the 2^−ΔΔCt^ method. Because *SiPIP1;5b* transcripts were not detected in wild-type plants (Ct > 35), expression levels among independent transgenic lines were normalized to the lowest-expressing transgenic line, which was assigned a value of 1. Primer sequences used for qRT-PCR are listed in Table S1.

### 4.5. Subcellular Localization

For transient expression assays, *A. tumefaciens* GV3101 carrying pCaMV35S::SiPIP1;5b-eGFP or pCaMV35S::eGFP was infiltrated into fully expanded leaves of 4–6-week-old *Nicotiana benthamiana plants*. After incubation under normal growth conditions for 48 h, GFP fluorescence was observed using a Leica TCS SP8 laser-scanning confocal microscope (Leica Microsystems, Wetzlar, Germany). GFP fluorescence was excited at 488 nm and detected at 505–530 nm. Bright-field images were simultaneously acquired and merged with fluorescence images. At least three leaves with independent infiltration were examined for each construct.

### 4.6. Freezing and Chilling Treatments

For freezing tolerance assays, four-week-old *Arabidopsis* plants grown at 22 °C were directly subjected to freezing treatment without any cold-acclimation pretreatment. The temperature in a programmable growth chamber was lowered from 22 °C to −4 °C at a cooling rate of −2 °C h^−1^, and the plants were then held at −4 °C for 4 h; ice nucleation occurred naturally, without artificial induction. The chamber temperature was subsequently raised back to 22 °C at +2 °C h^−1^, and plant phenotypes and survival were recorded after 3 d of recovery. Fully expanded leaves were collected immediately after the freezing treatment for physiological, biochemical, and histochemical analyses.

For chilling tolerance assays, five-week-old wild-type and transgenic tomato plants were exposed to 0 °C for 4 h, whereas plants maintained at 25 °C were used as controls. Fully expanded leaves were collected immediately after treatment for physiological, biochemical and histochemical analyses.

### 4.7. Physiological and Biochemical Measurements

Relative electrolyte conductivity was determined using a conductivity meter. Fresh leaf samples (0.1 g) were immersed in 10 mL of deionized water and vacuum infiltrated. After incubation at room temperature for 24 h, the initial conductivity (C_1_) was measured. The samples were subsequently boiled in a water bath for 30 min and cooled to room temperature, after which the final conductivity (C_2_) was recorded. Relative electrolyte conductivity was calculated as follows:

Relative electrolyte conductivity (%) = C_1_/C_2_ × 100

Malondialdehyde (MDA) content, superoxide dismutase (SOD) activity, peroxidase (POD) activity, catalase (CAT) activity, chlorophyll content, and free proline content were determined using commercial assay kits (Solarbio Science & Technology Co., Ltd., Beijing, China; Cat. Nos. BC0025, BC0175, BC0095, BC0205, BC0995, and BC0295, respectively) according to the manufacturer’s instructions.

The relative water content (RWC) of tomato leaves was determined gravimetrically. The fresh weight (FW) was recorded immediately after sampling. Leaf samples were fully hydrated to obtain turgid weight (TW) and subsequently dried to constant weight to determine dry weight (DW). RWC was calculated as follows:

RWC (%) = [(FW − DW)/(TW − DW)] × 100

### 4.8. Histochemical Detection of Reactive Oxygen Species

Reactive oxygen species accumulation was evaluated by histochemical staining using commercial kits (Solarbio, Beijing, China). Superoxide (O_2_•^−^) was detected with a nitroblue tetrazolium (NBT) staining kit (Cat. No. G4816), and hydrogen peroxide (H_2_O_2_) was detected with a 3,3′-diaminobenzidine (DAB) staining kit (Cat. No. G4815). Briefly, freshly collected leaves were immersed in NBT or DAB staining solution, subjected to vacuum infiltration at −0.1 MPa for 30 min, and then incubated at room temperature in the dark for 4–12 h until the positive areas appeared dark blue (NBT) or dark brown (DAB). Subsequently, the leaves were rinsed with distilled water and decolorized in the kit-supplied decolorization solution at 70–80 °C for 20–40 min to remove chlorophyll. The stained samples were then preserved in preservation solution and photographed under identical imaging conditions.

### 4.9. Field Experiment and Evaluation of Agronomic Traits

Field experiments were conducted in 2024 at the experimental field of Shihezi University, Shihezi, Xinjiang, China (44°18′ N, 86°03′ E), using T3-generation homozygous *SiPIP1;5b*-overexpressing tomato plants (line *SiPIP1;5b*-OE, i.e., the high-expression line OE-2) and wild-type *S. lycopersicum* cv. Yaxin87-5 plants. Seeds were sown on 2 April 2024 in a heated greenhouse, and the seedlings were transplanted into the field on 10 May 2024. The experiment was arranged as a randomized complete block design with three replicate blocks; each block contained one plot per genotype, and each plot was 15 m × 2 m. In each plot, one group of five consecutive plants per genotype was randomly selected for the measurements, so that 15 plants per genotype were evaluated in total (five plants per plot, i.e., per block). Plants were spaced 50 cm apart within rows, with 30 cm between rows, and were grown under drip irrigation with plastic-film mulching. All plots received identical management: irrigation every 7–10 d (approximately 300 m^3^ ha^−1^ per irrigation, eight irrigations in total during the growing season); basal fertilization before transplanting with 20 t ha^−1^ of well-decomposed organic manure and 300 kg ha^−1^ of a compound fertilizer (N:P_2_O_5_:K_2_O = 15:15:15), followed by topdressing with urea (150 kg ha^−1^) at the flowering and fruit-setting stages; manual weed control and routine pest and disease management were applied uniformly to all plots. Plants were pruned to a single stem and supported by stakes throughout the growing season.

At the fruit-ripening stage, plants were sampled from each plot for phenotypic and agronomic measurements, and plant height and stem diameter were measured at maturity. Fifteen plants were evaluated for each genotype (*n* = 15; five plants per plot, i.e., per block). The plot of origin was recorded for every plant, so that the block structure of the design could be included in the statistical analysis (Section 4.10 and Table S3). Six mature representative fruits were randomly harvested from each plant; fruit transverse and longitudinal diameters were measured, and the fruit shape index was calculated as the ratio of transverse diameter to longitudinal diameter. The fruit number per plant was recorded, and individual fruit weight was determined on the same six fruits. For fruit shape index and individual fruit weight, the mean value of the six fruits per plant was calculated first, and these plant-level means were used in the subsequent statistical analysis.

### 4.10. Statistical Analysis

All experiments were performed with at least three independent biological replicates, unless otherwise indicated. Data are presented as the mean ± standard error of the mean (SEM). Statistical analyses were performed using GraphPad Prism 10.4.1 (GraphPad Software, San Diego, CA, USA).

For physiological experiments involving genotype and temperature treatment as two factors, data were analyzed by two-way analysis of variance (ANOVA) followed by Dunnett’s multiple-comparison test, using the corresponding wild type as the reference genotype within each treatment group. For the field agronomic traits, the data were analysed in accordance with the randomized complete block design. In this design the experimental unit is the plot, and the five plants measured within a plot are subsamples; the plants sampled within a plot were therefore assigned to the corresponding block. Because the plot is the experimental unit, the primary inferential analysis was performed on plot means: the five plants of a plot were averaged first, and the three plot means per genotype were compared by a two-tailed Student’s *t*-test (*n* = 3 plots per genotype). This design-consistent analysis underlies all statements of significance, including the annotations in Figure 7. In addition, each trait was analysed by two-way ANOVA with genotype and block as fixed factors on the plant-level data (*n* = 15 plants per genotype), with the genotype effect tested against the residual mean square; this analysis accounts explicitly for the block structure and showed that the same conclusions are reached when the individual plants are analysed (Table S3). Values are reported as the mean ± SEM of the 15 plants measured per genotype (five plants per plot). Differences were considered statistically significant at *p* < 0.05.

## Data Availability

The original contributions presented in this study are included in the Supplementary Material. Further inquiries can be directed to the corresponding authors.

## Supplementary Materials

The following supporting information can be downloaded at: https://www.mdpi.com/article/10.3390/plants15203204/s1, Figure S1: Molecular identification and expression analysis of SiPIP1;5b-overexpressing Arabidopsis T1 lines; Figure S2: Genetic transformation, identification and expression analysis of SiPIP1;5b-overexpressing tomato; Table S1: Primer sequences used in this study; Table S2: Accession numbers and gene identifiers of the aquaporin sequences used for the phylogenetic analyses in Figure 1 and Figure 2; Table S3: Analysis of the agronomic traits of wild-type and SiPIP1;5b-overexpressing tomato plants in the 2024 field trial.

## References

[1] Jan S., Rustgi S., Barmukh R., Shikari A.B., Leske B., Bekuma A., Sharma D., Ma W., Kumar U., Kumar U. (2024). Advances and Opportunities in Unraveling Cold-Tolerance Mechanisms in the World’s Primary Staple Food Crops. Plant Genome.

[2] Thomashow M.F. (1999). Plant Cold Acclimation: Freezing Tolerance Genes and Regulatory Mechanisms. Annu. Rev. Plant Physiol. Plant Mol. Biol..

[3] Vyse K., Penzlin J., Sergeant K., Hincha D.K., Arora R., Zuther E. (2020). Repair of Sub-Lethal Freezing Damage in Leaves of *Arabidopsis thaliana*. BMC Plant Biol..

[4] Kutsuno T., Chowhan S., Kotake T., Takahashi D. (2023). Temporal Cell Wall Changes during Cold Acclimation and Deacclimation and Their Potential Involvement in Freezing Tolerance and Growth. Physiol. Plant..

[5] Wei M., Liu X., Wang Y., Yan G., Li Q., Tang C., Shi F. (2026). Molecular Mechanisms of Plant Freezing Tolerance: From Cold Signal Perception to Adaptive Responses. Front. Plant Sci..

[6] Hu Y., Tian C., Song S., Li R. (2024). Insights on the Enhancement of Chilling Tolerance in Rice through Over-Expression and Knock-out Studies of *OsRBCS3*. Plant Signal. Behav..

[7] Maurel C., Verdoucq L., Luu D.-T., Santoni V. (2008). Plant Aquaporins: Membrane Channels with Multiple Integrated Functions. Annu. Rev. Plant Biol..

[8] Maurel C., Boursiac Y., Luu D., Santoni V., Shahzad Z., Verdoucq L. (2015). Aquaporins in Plants. Physiol. Rev..

[9] Verdoucq L., Rodrigues O., Martinière A., Luu D.T., Maurel C. (2014). Plant Aquaporins on the Move: Reversible Phosphorylation, Lateral Motion and Cycling. Curr. Opin. Plant Biol..

[10] Kaldenhoff R., Fischer M. (2006). Aquaporins in Plants. Acta Physiol..

[11] Jang J.Y., Kim D.G., Kim Y.O., Kim J.S., Kang H. (2004). An Expression Analysis of a Gene Family Encoding Plasma Membrane Aquaporins in Response to Abiotic Stresses in *Arabidopsis thaliana*. Plant Mol. Biol..

[12] Johanson U., Karlsson M., Johansson I., Gustavsson S., Sjövall S., Fraysse L., Weig A.R., Kjellbom P. (2001). The Complete Set of Genes Encoding Major Intrinsic Proteins in *Arabidopsis* Provides a Framework for a New Nomenclature for Major Intrinsic Proteins in Plants. Plant Physiol..

[13] Liu J., Qi W., Lu H., Shao H., Zhang D. (2021). Characterization of Interactions between the Soybean Salt-Stress Responsive Membrane-Intrinsic Proteins *GmPIP1* and *GmPIP2*. Agronomy.

[14] Alexandersson E., Danielson J.Å.H., Råde J., Moparthi V.K., Fontes M., Kjellbom P., Johanson U. (2010). Transcriptional Regulation of Aquaporins in Accessions of *Arabidopsis* in Response to Drought Stress. Plant J..

[15] Singh R.K., Deshmukh R., Muthamilarasan M., Rani R., Prasad M. (2020). Versatile Roles of Aquaporin in Physiological Processes and Stress Tolerance in Plants. Plant Physiol. Biochem..

[16] Yepes-Molina L., Bárzana G., Carvajal M. (2020). Controversial Regulation of Gene Expression and Protein Transduction of Aquaporins under Drought and Salinity Stress. Plants.

[17] Rahman A., Kawamura Y., Maeshima M., Rahman A., Uemura M. (2020). Plasma Membrane Aquaporin Members *PIPs* Act in Concert to Regulate Cold Acclimation and Freezing Tolerance Responses in *Arabidopsis thaliana*. Plant Cell Physiol..

[18] Li G.-W., Zhang M.-H., Cai W.-M., Sun W.-N., Su W.-A. (2008). Characterization of *OsPIP2;7*, a Water Channel Protein in Rice. Plant Cell Physiol..

[19] Zhuo C., Wang T., Guo Z., Lu S. (2016). Overexpression of *MfPIP2-7* from *Medicago falcata* Promotes Cold Tolerance and Growth under NO_3_^−^ Deficiency in Transgenic Tobacco Plants. BMC Plant Biol..

[20] Fei J., Wang Y., Cheng H., Wang H., Wu M., Sun F., Sun C. (2023). An Aquaporin Gene (*KoPIP2;1*) Isolated from Mangrove Plant *Kandelia Obovata* Had Enhanced Cold Tolerance of Transgenic *Arabidopsis thaliana*. Bioengineering.

[21] Han Y., Luo F., Liang A., Xu D., Zhang H., Liu T., Qi H. (2024). Aquaporin *CmPIP2;3* Links H_2_O_2_ Signal and Antioxidation to Modulate Trehalose-Induced Cold Tolerance in Melon Seedlings. Plant Physiol..

[22] Jang J.Y., Lee S.H., Rhee J.Y., Chung G.C., Ahn S.J., Kang H. (2007). Transgenic *Arabidopsis* and Tobacco Plants Overexpressing an Aquaporin Respond Differently to Various Abiotic Stresses. Plant Mol. Biol..

[23] He W.-D., Gao J., Dou T.-X., Shao X.-H., Bi F.-C., Sheng O., Deng G.-M., Li C.-Y., Hu C.-H., Liu J.-H. (2018). Early Cold-Induced Peroxidases and Aquaporins Are Associated With High Cold Tolerance in Dajiao (*Musa* spp. ‘Dajiao’). Front. Plant Sci..

[24] Li J., Liu H., Xia W., Mu J., Feng Y., Liu R., Yan P., Wang A., Lin Z., Guo Y. (2017). De Novo Transcriptome Sequencing and the Hypothetical Cold Response Mode of *Saussurea involucrata* in Extreme Cold Environments. Int. J. Mol. Sci..

[25] Mu J., Fu Y., Liu B., Zhang Y., Wang A., Li Y., Zhu J. (2021). *SiFBA5*, a Cold-Responsive Factor from *Saussurea involucrata* Promotes Cold Resilience and Biomass Increase in Transgenic Tomato Plants under Cold Stress. BMC Plant Biol..

[26] Guo X., Zhang L., Wang X., Zhang M., Xi Y., Wang A., Zhu J. (2019). Overexpression of *Saussurea involucrata* Dehydrin Gene *SiDHN* Promotes Cold and Drought Tolerance in Transgenic Tomato Plants. PLoS ONE.

[27] Liu X., Xia W., Zhang X., Li A., Qin J., Sun H., Li J., Zhu J. (2022). Overexpression of the *SiLEA5* Gene in *Saussurea involucrata* Increases the Low-Temperature Tolerance of Transgenic Tomatoes. Horticulturae.

[28] Liu X., Xia W., Zhang D., Li A., Li J., Zhu J. (2023). Cold Tolerance Gene *SiLEA* B19.3 of *Saussurea involucrata* Increases the Yield of Transgenic Tomato. S. Afr. J. Bot..

[29] Li J., Xia W., Zang H., Dai B., Zhang Y., Feng Y., Wang A., Lin Z., Liu H., Zhu J. (2020). Expression Analysis of Aquaporin Genes in *Saussurea involucrata* Rosette Leaves and Functional Analysis of Upregulated *SiPIP1;5A* under Low-Temperature Stress. Environ. Exp. Bot..

[30] Li R., Zhang L., Wang L., Chen L., Zhao R., Sheng J., Shen L. (2018). Reduction of Tomato-Plant Chilling Tolerance by CRISPR–Cas9-Mediated *SlCBF1* Mutagenesis. J. Agric. Food Chem..

[31] Chaumont F., Tyerman S.D. (2014). Aquaporins: Highly Regulated Channels Controlling Plant Water Relations. Plant Physiol..

[32] Peng Y., Arora R., Li G., Wang X., Fessehaie A. (2008). *Rhododendron catawbiense* Plasma Membrane Intrinsic Proteins Are Aquaporins, and Their Over-Expression Compromises Constitutive Freezing Tolerance and Cold Acclimation Ability of Transgenic *Arabidopsis* Plants. Plant Cell Environ..

[33] Bienert G.P., Møller A.L.B., Kristiansen K.A., Schulz A., Møller I.M., Schjoerring J.K., Jahn T.P. (2007). Specific Aquaporins Facilitate the Diffusion of Hydrogen Peroxide across Membranes. J. Biol. Chem..

[34] Dynowski M., Schaaf G., Loque D., Moran O., Ludewig U. (2008). Plant Plasma Membrane Water Channels Conduct the Signalling Molecule H_2_O_2_. Biochem. J..

[35] Rodrigues O., Reshetnyak G., Grondin A., Saijo Y., Leonhardt N., Maurel C., Verdoucq L. (2017). Aquaporins Facilitate Hydrogen Peroxide Entry into Guard Cells to Mediate ABA- and Pathogen-Triggered Stomatal Closure. Proc. Natl. Acad. Sci. USA.

[36] Israel D., Lee S.H., Robson T.M., Zwiazek J.J. (2022). Plasma Membrane Aquaporins of the *PIP1* and *PIP2* Subfamilies Facilitate Hydrogen Peroxide Diffusion into Plant Roots. BMC Plant Biol..

[37] Chevriau J., De Palma G.Z., Jozefkowicz C., Vitali V., Canessa Fortuna A., Ayub N., Soto G., Bienert G.P., Zeida A., Alleva K. (2024). Permeation Mechanisms of Hydrogen Peroxide and Water through Plasma Membrane Intrinsic Protein Aquaporins. Biochem. J..

[38] Alam M.M., Rafi A., Rahman M.A., Mitu M.A., Sakib M.N., Rahman M.A., Rahimi M., Kordrostami M., Biswas A., Rashad M.M.I. (2026). Aquaporins as Natural Stress Integrator: Coordinating Transport, Signals, and Tolerance Mechanisms in Plants. Plant Cell Environ..

[39] Czechowski T., Stitt M., Altmann T., Udvardi M.K., Scheible W.-R. (2005). Genome-Wide Identification and Testing of Superior Reference Genes for Transcript Normalization in Arabidopsis. Plant Physiol..

[40] Expósito-Rodríguez M., Borges A.A., Borges-Pérez A., Pérez J.A. (2008). Selection of Internal Control Genes for Quantitative Real-Time RT-PCR Studies during Tomato Development Process. BMC Plant Biol..

