# Supplementary material for: Heterologous Expression of Saussurea involucrata SiPIP1;5b Enhances Freezing Tolerance in Arabidopsis and Chilling Tolerance in Tomato
Source: Plants (Basel). 2026 Oct 19;15(20):3204. doi: 10.3390/plants15203204 (PMC13709195; doi:10.3390/plants15203204)
Supplement: Supplementary file 1 [file plants-15-03204-s001.zip › plants-4569287-supplementary.pdf]

Supplementary Materials: Figure S1; Figure S2; Table S1; Table S2; Table S3.

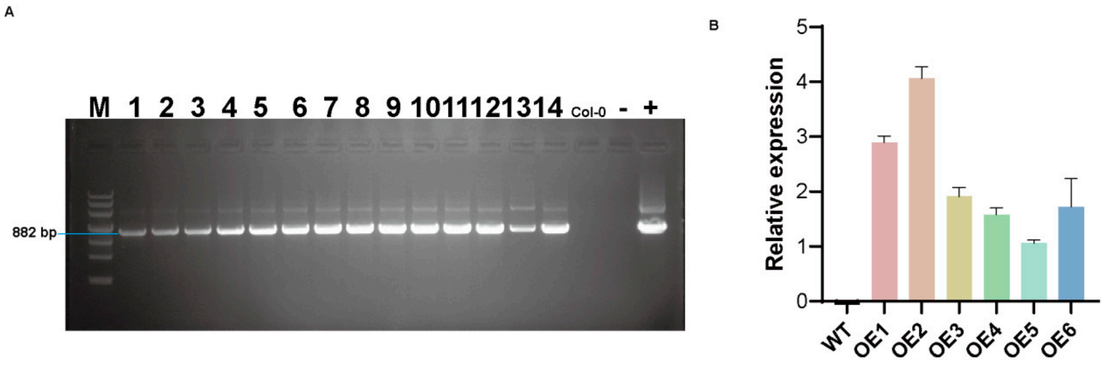

Figure S1. Molecular identification and expression analysis of *SiPIP1;5b*-overexpressing *Arabidopsis* T1 lines. (A) PCR identification of T1 transgenic lines. M, DNA Marker III; lanes 1–14, independent T1 lines; Col-0, wild type; −, blank control; +, plasmid positive control. The target fragment is 882 bp. (B) Relative expression of *SiPIP1;5b* in transgenic lines by qRT-PCR (*AtACT2* as internal reference). No exogenous expression was detected in WT (Not Detected). The line with the lowest expression was used as calibrator (= 1), and relative expression was calculated by the 2^(−ΔΔCt) method. Lines OE-1–OE-3 showed high expression and were used for subsequent experiments. Data are means ± SEM (n = 3).

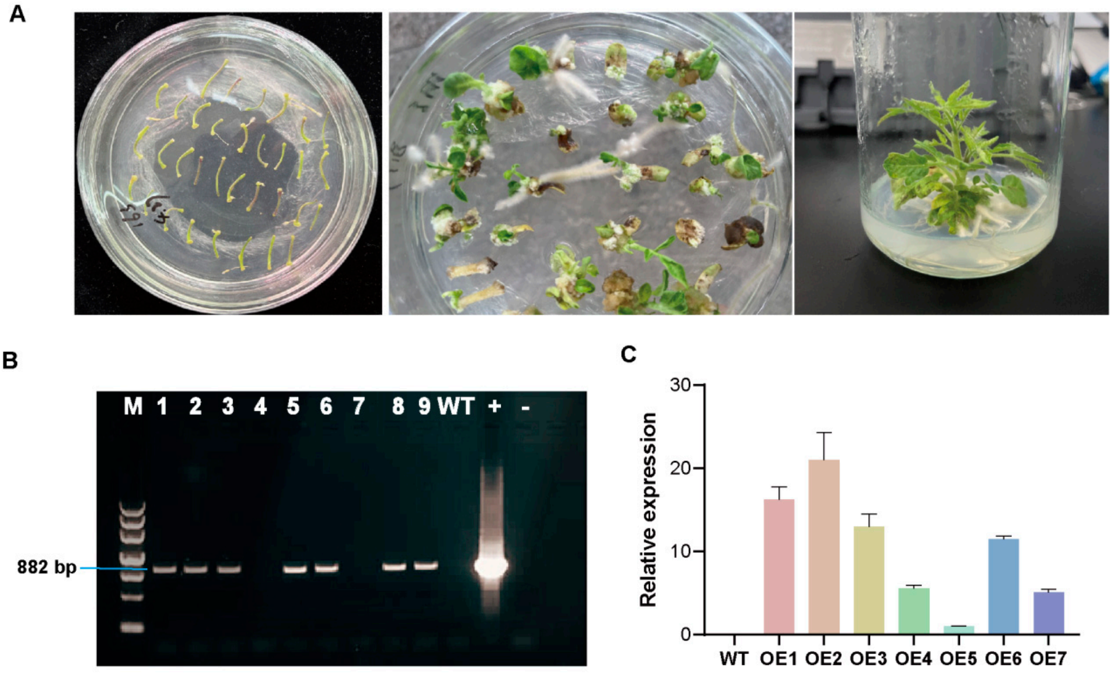

Figure S2. Genetic transformation, identification and expression analysis of *SiPIP1;5b*-overexpressing tomato. (A) Tomato genetic transformation and plant regeneration. Left, cotyledon explants after co-cultivation; middle, adventitious shoots from resistant calli; right, rooted regenerated plantlet. (B) PCR identification of T0 transgenic tomato lines. M, DNA Marker; lanes 1–9, independent resistant lines; WT, wild type; +, plasmid positive control; −, blank control. The target fragment is 882 bp, and seven positive lines (OE-1–OE7) were obtained. (C) Relative expression of *SiPIP1;5b* in transgenic tomato lines by qRT-PCR (*SlACT* as internal reference). No exogenous expression was detected in WT (Not Detected). The line with the lowest expression was used as calibrator (= 1), and relative expression was calculated by the 2^(−ΔΔCt) method. Lines OE-1–OE-3 showed high expression and were used for subsequent experiments. Data are means ± SEM (n = 3).

Table S1. Primer sequences used in this study. *AtACT2* and *SlACT* were used as the internal reference genes for *Arabidopsis* and tomato, respectively; *AtACT7* primers were retained as an alternative reference gene and were not used in the present study.

Primer	Sequence (5′→3′)	Purpose
SiPIP1;5b-EcoRI-F	gaattcATGGAGGGTAAGGAAGAAGATGTG	Gene cloning
SiPIP1;5b-KpnI-R	ggtaccTTAATAATGAATATTATTTTTGATCTGCTCTT-GAA	Gene cloning
SiPIP1;5b-qF	GCATCAACCCTGCAAGAAGT	qPCR
SiPIP1;5b-qR	GATACACAGCAGCAAGTGCA	qPCR
AtACT2-qF	CTCAGGTATCGCTGACCGTA	qPCR (internal control, Arabidopsis)
AtACT2-qR	AGAATGGAACCACCGATCCA	qPCR (internal control, Arabidopsis)
AtACT7-qF	CTCTCTATGCCAGTGGTCGTA	qPCR (alternative reference gene, not used in this study)
AtACT7-qR	CAAGATCAAGACGAAGGATAGCAT	qPCR (alternative reference gene, not used in this study)
SlACT-qF	GCAATTGGGATGATATGGAGAAGA	qPCR (internal control, tomato)
SlACT-qR	CACGATTAGCCTTTGGGTTAAGA	qPCR (internal control, tomato)

Table S2. Accession numbers and gene identifiers of the aquaporin sequences used for the phylogenetic analyses in Figures 1 and 2. Sequences of *Arabidopsis thaliana*, *Oryza sativa* and *Helianthus annuus* were retrieved from UniProtKB (release 2026_03); the 33 *Saussurea involucrata* sequences were obtained from the in-house genome/transcriptome assembly of this laboratory (transcript IDs asmbl_XXXXX.p1). Gene structures in Figure 2 were derived from the same 33 *S. involucrata* sequences.

Species	Name used in this study (Figures 1, 2)	Accession number / gene identifier	Database / source	Length (aa)	Genomic location	Notes
Saussurea involucrata	SiNIP1;2a	asmbl_94073.p1	In-house assembly (this study)	215	CM066325.1: 73863810-73868966 (- strand), 5157 bp	exons 5 / introns 4; orthologue AtNIP1;2 (70.6% identity); gene ID asmbl_94073
Saussurea involucrata	SiNIP1;2b	asmbl_94072.p1	In-house assembly (this study)	285	CM066325.1: 73863778-73868967 (- strand), 5190 bp	exons 5 / introns 4; orthologue AtNIP1;2 (69.2% identity); gene ID asmbl_94072
Saussurea involucrata	SiNIP1;2c	asmbl_2988.p1	In-house assembly (this study)	210	CM066312.1: 93203449-93205385 (- strand), 1937 bp	exons 3 / introns 2; orthologue AtNIP1;2

Species	Name used in this study (Figures 1, 2)	Accession number / gene identifier	Database / source	Length (aa)	Genomic location	Notes
						(56.6% identity); gene ID asmbl_2988
Saussurea involucrata	SiNIP4;2a	asmbl_73374.p1	In-house assembly (this study)	194	CM066322.1: 54840173-54841685 (+ strand), 1513 bp	exons 4 / introns 3; orthologue AtNIP4;2 (59.8% identity); gene ID asmbl_73374
Saussurea involucrata	SiNIP4;2b	asmbl_73375.p1	In-house assembly (this study)	255	CM066322.1: 54840196-54841685 (+ strand), 1490 bp	exons 5 / introns 4; orthologue AtNIP4;2 (59.6% identity); gene ID asmbl_73375
Saussurea involucrata	SiNIP5;1	asmbl_62230.p1	In-house assembly (this study)	295	CM066321.1: 144976898-144978842 (- strand), 1945 bp	exons 4 / introns 3; orthologue AtNIP5;1 (80.5% identity); gene ID asmbl_62230
Saussurea involucrata	SiPIP1;3a	asmbl_8727.p1	In-house assembly (this study)	288	CM066313.1: 103164960-103168809 (- strand), 3850 bp	exons 4 / introns 3; orthologue AtPIP1;3 (90.9% identity); gene ID asmbl_8727
Saussurea involucrata	SiPIP1;5a	asmbl_61654.p1	In-house assembly (this study)	301	CM066321.1: 139777957-139780934 (+ strand), 2978 bp	exons 4 / introns 3; orthologue AtPIP1;1 (88.5% identity); gene ID asmbl_61654
Saussurea involucrata	SiPIP1;5b	asmbl_57966.p1	In-house assembly (this study)	293	CM066321.1: 95906937-95908863 (+ strand), 1927 bp	exons 4 / introns 3; orthologue AtPIP1;3 (90.6% identity); gene ID asmbl_57966
Saussurea involucrata	SiPIP2;1a	asmbl_70467.p1	In-house assembly (this study)	308	CM066322.1: 29182845-29184393 (- strand), 1549 bp	exons 4 / introns 3; orthologue AtPIP2;1 (83.2% identity); gene ID asmbl_70467
Saussurea involucrata	SiPIP2;1b	asmbl_70464.p1	In-house assembly (this study)	283	CM066322.1: 29071428-29072886 (+ strand), 1459 bp	exons 4 / introns 3; orthologue AtPIP2;1 (80.7% identity); gene ID asmbl_70464
Saussurea involucrata	SiPIP2;2a	asmbl_91205.p1	In-house assembly (this study)	306	CM066325.1: 15385627-15387782 (+ strand), 2156 bp	exons 4 / introns 3; orthologue AtPIP2;2 (87.7% identity); gene ID asmbl_91205

Species	Name used in this study (Figures 1, 2)	Accession number / gene identifier	Database / source	Length (aa)	Genomic location	Notes
Saussurea involucrata	SiPIP2;2b	asmbl_69619.p1	In-house assembly (this study)	322	CM066322.1: 21274185-21275560 (- strand), 1376 bp	exons 3 / introns 2; orthologue AtPIP2;2 (84.0% identity); gene ID asmbl_69619
Saussurea involucrata	SiPIP2;2c	asmbl_22336.p1	In-house assembly (this study)	292	CM066315.1: 41360287-41362202 (+ strand), 1916 bp	exons 4 / introns 3; orthologue AtPIP2;2 (80.6% identity); gene ID asmbl_22336
Saussurea involucrata	SiPIP2;4a	asmbl_71177.p1	In-house assembly (this study)	290	CM066322.1: 35778707-35780965 (- strand), 2259 bp	exons 4 / introns 3; orthologue AtPIP2;4 (84.9% identity); gene ID asmbl_71177
Saussurea involucrata	SiPIP2;4b	asmbl_70463.p1	In-house assembly (this study)	306	CM066322.1: 29071423-29072880 (+ strand), 1458 bp	exons 4 / introns 3; orthologue AtPIP2;4 (83.6% identity); gene ID asmbl_70463
Saussurea involucrata	SiPIP2;4c	asmbl_70465.p1	In-house assembly (this study)	278	CM066322.1: 29117834-29120234 (- strand), 2401 bp	exons 4 / introns 3; orthologue AtPIP2;4 (78.5% identity); gene ID asmbl_70465
Saussurea involucrata	SiPIP2;8a	asmbl_64649.p1	In-house assembly (this study)	279	CM066321.1: 173219993-173221664 (+ strand), 1672 bp	exons 4 / introns 3; orthologue AtPIP2;8 (90.6% identity); gene ID asmbl_64649
Saussurea involucrata	SiPIP2;8b	asmbl_70466.p1	In-house assembly (this study)	194	CM066322.1: 29117791-29120180 (- strand), 2390 bp	exons 4 / introns 3; orthologue AtPIP2;8 (76.3% identity); gene ID asmbl_70466
Saussurea involucrata	SiSIP1;1a	asmbl_90839.p1	In-house assembly (this study)	236	CM066325.1: 11750243-11753209 (+ strand), 2967 bp	exons 3 / introns 2; orthologue AtSIP1;1 (59.2% identity); gene ID asmbl_90839
Saussurea involucrata	SiSIP1;1b	asmbl_60988.p1	In-house assembly (this study)	248	CM066321.1: 133082117-133083208 (+ strand), 1092 bp	exons 3 / introns 2; orthologue AtSIP1;1 (52.6% identity); gene ID asmbl_60988
Saussurea involucrata	SiSIP2;1a	asmbl_99728.p1	In-house assembly (this study)	230	CM066327.1: 10410475-10413177 (- strand), 2703 bp	exons 3 / introns 2; orthologue AtSIP2;1 (54.6% identity); gene ID asmbl_99728

Species	Name used in this study (Figures 1, 2)	Accession number / gene identifier	Database / source	Length (aa)	Genomic location	Notes
Saussurea involucrata	SiSIP2;1b	asmbl_99730.p1	In-house assembly (this study)	212	CM066327.1: 10410920-10413159 (- strand), 2240 bp	exons 2 / introns 1; orthologue AtSIP2;1 (49.8% identity); gene ID asmbl_99730
Saussurea involucrata	SiTIP1;1a	asmbl_90248.p1	In-house assembly (this study)	263	CM066325.1: 6829217-6830474 (+ strand), 1258 bp	exons 2 / introns 1; orthologue AtTIP1;1 (81.2% identity); gene ID asmbl_90248
Saussurea involucrata	SiTIP1;1b	asmbl_91075.p1	In-house assembly (this study)	257	CM066325.1: 13363117-13364516 (+ strand), 1400 bp	exons 2 / introns 1; orthologue AtTIP1;1 (75.7% identity); gene ID asmbl_91075
Saussurea involucrata	SiTIP1;1c	asmbl_60727.p1	In-house assembly (this study)	250	CM066321.1: 130266764-130268358 (- strand), 1595 bp	exons 2 / introns 1; orthologue AtTIP1;1 (71.1% identity); gene ID asmbl_60727
Saussurea involucrata	SiTIP2;1a	asmbl_9841.p1	In-house assembly (this study)	248	CM066314.1: 5725780-5727803 (+ strand), 2024 bp	exons 3 / introns 2; orthologue AtTIP2;1 (83.5% identity); gene ID asmbl_9841
Saussurea involucrata	SiTIP2;1b	asmbl_39199.p1	In-house assembly (this study)	248	CM066318.1: 121854872-121856179 (+ strand), 1308 bp	exons 3 / introns 2; orthologue AtTIP2;1 (80.2% identity); gene ID asmbl_39199
Saussurea involucrata	SiTIP2;2a	asmbl_56522.p1	In-house assembly (this study)	250	CM066321.1: 68537867-68539281 (- strand), 1415 bp	exons 3 / introns 2; orthologue AtTIP2;2 (82.4% identity); gene ID asmbl_56522
Saussurea involucrata	SiTIP2;2b	asmbl_9065.p1	In-house assembly (this study)	251	CM066313.1: 106522613-106523818 (+ strand), 1206 bp	exons 3 / introns 2; orthologue AtTIP2;2 (81.6% identity); gene ID asmbl_9065
Saussurea involucrata	SiTIP3;1	asmbl_40262.p1	In-house assembly (this study)	257	CM066319.1: 2765311-2767963 (+ strand), 2653 bp	exons 3 / introns 2; orthologue AtTIP3;1 (69.0% identity); gene ID asmbl_40262
Saussurea involucrata	SiTIP3;2	asmbl_50559.p1	In-house assembly (this study)	319	CM066320.1: 133721362-133723189 (+ strand), 1828 bp	exons 3 / introns 2; orthologue AtTIP3;2 (30.5% identity); gene ID asmbl_50559

Species	Name used in this study (Figures 1, 2)	Accession number / gene identifier	Database / source	Length (aa)	Genomic location	Notes
Saussurea involucrata	SiTIP4;1	asmbl_58775.p1	In-house assembly (this study)	248	CM066321.1: 109778162-109780275 (+ strand), 2114 bp	exons 3 / introns 2; orthologue AtTIP4;1 (73.3% identity); gene ID asmbl_58775
Arabidopsis thaliana	AtNIP1	Q8GT75	UniProtKB (Swiss-Prot) 2026_03	236		gene name: NIP1; family: NIP
Arabidopsis thaliana	AtNIP1;1	Q8VZW1	UniProtKB (Swiss-Prot) 2026_03	296		gene name: NIP1-1; family: NIP
Arabidopsis thaliana	AtNIP1;2	Q8LFP7	UniProtKB (Swiss-Prot) 2026_03	294		gene name: NIP1-2; family: NIP
Arabidopsis thaliana	AtNIP2	Q8GT74	UniProtKB (Swiss-Prot) 2026_03	241		gene name: NIP2; family: NIP
Arabidopsis thaliana	AtNIP2;1	Q8W037	UniProtKB (Swiss-Prot) 2026_03	288		gene name: NIP2-1; family: NIP
Arabidopsis thaliana	AtNIP3;1	Q9C6T0	UniProtKB (Swiss-Prot) 2026_03	323		gene name: NIP3-1; family: NIP
Arabidopsis thaliana	AtNIP4;1	Q9FIZ9	UniProtKB (Swiss-Prot) 2026_03	283		gene name: NIP4-1; family: NIP
Arabidopsis thaliana	AtNIP4;2	Q8W036	UniProtKB (Swiss-Prot) 2026_03	283		gene name: NIP4-2; family: NIP
Arabidopsis thaliana	AtNIP5;1	Q9SV84	UniProtKB (Swiss-Prot) 2026_03	304		gene name: NIP5-1; family: NIP
Arabidopsis thaliana	AtNIP6;1	Q9SAI4	UniProtKB (Swiss-Prot) 2026_03	305		gene name: NIP6-1; family: NIP
Arabidopsis thaliana	AtNIP7;1	Q8LAI1	UniProtKB (Swiss-Prot) 2026_03	275		gene name: NIP7-1; family: NIP
Arabidopsis thaliana	AtPIP1;1	P61837	UniProtKB (Swiss-Prot) 2026_03	286		gene name: PIP1-1; family: PIP
Arabidopsis thaliana	AtPIP1;2	Q06611	UniProtKB (Swiss-Prot) 2026_03	286		gene name: PIP1-2; family: PIP
Arabidopsis thaliana	AtPIP1;3	Q08733	UniProtKB (Swiss-Prot) 2026_03	286		gene name: PIP1-3; family: PIP
Arabidopsis thaliana	AtPIP1;4	Q39196	UniProtKB (Swiss-Prot) 2026_03	287		gene name: PIP1.4; family: PIP
Arabidopsis thaliana	AtPIP1;5	Q8LAA6	UniProtKB (Swiss-Prot) 2026_03	287		gene name: PIP1-5; family: PIP
Arabidopsis thaliana	AtPIP2;1	P43286	UniProtKB (Swiss-Prot) 2026_03	287		gene name: PIP2-1; family: PIP
Arabidopsis thaliana	AtPIP2;2	P43287	UniProtKB (Swiss-Prot) 2026_03	285		gene name: PIP2-2; family: PIP

Species	Name used in this study (Figures 1, 2)	Accession number / gene identifier	Database / source	Length (aa)	Genomic location	Notes
Arabidopsis thaliana	AtPIP2;3	P30302	UniProtKB (Swiss-Prot) 2026_03	285		gene name: PIP2-3; family: PIP
Arabidopsis thaliana	AtPIP2;4	Q9FF53	UniProtKB (Swiss-Prot) 2026_03	291		gene name: PIP2-4; family: PIP
Arabidopsis thaliana	AtPIP2;5	Q9SV31	UniProtKB (Swiss-Prot) 2026_03	286		gene name: PIP2-5; family: PIP
Arabidopsis thaliana	AtPIP2;6	Q9ZV07	UniProtKB (Swiss-Prot) 2026_03	289		gene name: PIP2-6; family: PIP
Arabidopsis thaliana	AtPIP2;7	P93004	UniProtKB (Swiss-Prot) 2026_03	280		gene name: PIP2-7; family: PIP
Arabidopsis thaliana	AtPIP2;8	Q9ZVX8	UniProtKB (Swiss-Prot) 2026_03	278		gene name: PIP2-8; family: PIP
Arabidopsis thaliana	AtSIP1;1	Q9M8W5	UniProtKB (Swiss-Prot) 2026_03	240		gene name: SIP1-1; family: SIP
Arabidopsis thaliana	AtSIP1;2	Q9FK43	UniProtKB (Swiss-Prot) 2026_03	243		gene name: SIP1-2; family: SIP
Arabidopsis thaliana	AtSIP2;1	Q9M1K3	UniProtKB (Swiss-Prot) 2026_03	237		gene name: SIP2-1; family: SIP
Arabidopsis thaliana	AtTIP1;1	P25818	UniProtKB (Swiss-Prot) 2026_03	251		gene name: TIP1-1; family: TIP
Arabidopsis thaliana	AtTIP1;2	Q41963	UniProtKB (Swiss-Prot) 2026_03	253		gene name: TIP1-2; family: TIP
Arabidopsis thaliana	AtTIP1;3	O82598	UniProtKB (Swiss-Prot) 2026_03	252		gene name: TIP1-3; family: TIP
Arabidopsis thaliana	AtTIP2;1	Q41951	UniProtKB (Swiss-Prot) 2026_03	250		gene name: TIP2-1; family: TIP
Arabidopsis thaliana	AtTIP2;2	Q41975	UniProtKB (Swiss-Prot) 2026_03	250		gene name: TIP2-2; family: TIP
Arabidopsis thaliana	AtTIP2;3	Q9FGL2	UniProtKB (Swiss-Prot) 2026_03	250		gene name: TIP2-3; family: TIP
Arabidopsis thaliana	AtTIP3;1	P26587	UniProtKB (Swiss-Prot) 2026_03	268		gene name: TIP3-1; family: TIP
Arabidopsis thaliana	AtTIP3;2	O22588	UniProtKB (Swiss-Prot) 2026_03	267		gene name: TIP3-2; family: TIP
Arabidopsis thaliana	AtTIP41L	Q8VXY4	UniProtKB (Swiss-Prot) 2026_03	290		gene name: TIP41L; family: TIP
Arabidopsis thaliana	AtTIP4;1	O82316	UniProtKB (Swiss-Prot) 2026_03	249		gene name: TIP4-1; family: TIP
Arabidopsis thaliana	AtTIP5;1	Q9STX9	UniProtKB (Swiss-Prot) 2026_03	256		gene name: TIP5-1; family: TIP

Species	Name used in this study (Figures 1, 2)	Accession number / gene identifier	Database / source	Length (aa)	Genomic location	Notes
Oryza sativa	OsNIP1;1	Q40746	UniProtKB (Swiss-Prot) 2026_03	284		gene name: NIP1-1; family: NIP
Oryza sativa	OsNIP1;2	Q0JPT5	UniProtKB (Swiss-Prot) 2026_03	303		gene name: NIP1-2; family: NIP
Oryza sativa	OsNIP1;3	Q0DK16	UniProtKB (Swiss-Prot) 2026_03	286		gene name: NIP1-3; family: NIP
Oryza sativa	OsNIP1;4	Q5Z9E2	UniProtKB (Swiss-Prot) 2026_03	273		gene name: NIP1-4; family: NIP
Oryza sativa	OsNIP2;1	Q6Z2T3	UniProtKB (Swiss-Prot) 2026_03	298		gene name: NIP2-1; family: NIP
Oryza sativa	OsNIP2;2	Q67WJ8	UniProtKB (Swiss-Prot) 2026_03	298		gene name: NIP2-2; family: NIP
Oryza sativa	OsNIP3;1	Q0IWF3	UniProtKB (Swiss-Prot) 2026_03	311		gene name: NIP3-1; family: NIP
Oryza sativa	OsNIP3;2	Q7EYH7	UniProtKB (Swiss-Prot) 2026_03	305		gene name: NIP3-2; family: NIP
Oryza sativa	OsNIP3;3	Q84S07	UniProtKB (Swiss-Prot) 2026_03	278		gene name: NIP3-3; family: NIP
Oryza sativa	OsNIP4;1	Q9ASI1	UniProtKB (Swiss-Prot) 2026_03	286		gene name: NIP4-1; family: NIP
Oryza sativa	OsPIP1;1	Q6EU94	UniProtKB (Swiss-Prot) 2026_03	289		gene name: PIP1-1; family: PIP
Oryza sativa	OsPIP1;2	Q7XSQ9	UniProtKB (Swiss-Prot) 2026_03	288		gene name: PIP1-2; family: PIP
Oryza sativa	OsPIP1;3	Q9SXF8	UniProtKB (Swiss-Prot) 2026_03	288		gene name: PIP1-3; family: PIP
Oryza sativa	OsPIP2;1	Q8H5N9	UniProtKB (Swiss-Prot) 2026_03	290		gene name: PIP2-1; family: PIP
Oryza sativa	OsPIP2;2	Q6K215	UniProtKB (Swiss-Prot) 2026_03	288		gene name: PIP2-2; family: PIP
Oryza sativa	OsPIP2;3	Q7XUA6	UniProtKB (Swiss-Prot) 2026_03	290		gene name: PIP2-3; family: PIP
Oryza sativa	OsPIP2;4	Q8GRT8	UniProtKB (Swiss-Prot) 2026_03	286		gene name: PIP2-4; family: PIP
Oryza sativa	OsPIP2;5	Q8GRI8	UniProtKB (Swiss-Prot) 2026_03	283		gene name: PIP2-5; family: PIP
Oryza sativa	OsPIP2;6	Q7XLR1	UniProtKB (Swiss-Prot) 2026_03	282		gene name: PIP2-6; family: PIP
Oryza sativa	OsPIP2;7	Q651D5	UniProtKB (Swiss-Prot) 2026_03	290		gene name: PIP2-7; family: PIP

Species	Name used in this study (Figures 1, 2)	Accession number / gene identifier	Database / source	Length (aa)	Genomic location	Notes
Oryza sativa	OsPIP2;8	Q7Y1E6	UniProtKB (Swiss-Prot) 2026_03	280		gene name: PIP2-8; family: PIP
Oryza sativa	OsSIP1;1	Q5VR89	UniProtKB (Swiss-Prot) 2026_03	246		gene name: SIP1-1; family: SIP
Oryza sativa	OsSIP2;1	Q10M80	UniProtKB (Swiss-Prot) 2026_03	250		gene name: SIP2-1; family: SIP
Oryza sativa	OsTIP1;1	P50156	UniProtKB (Swiss-Prot) 2026_03	250		gene name: TIP1-1; family: TIP
Oryza sativa	OsTIP1;2	Q94CS9	UniProtKB (Swiss-Prot) 2026_03	252		gene name: TIP1-2; family: TIP
Oryza sativa	OsTIP2;1	Q7XA61	UniProtKB (Swiss-Prot) 2026_03	248		gene name: TIP2-1; family: TIP
Oryza sativa	OsTIP2;2	Q5Z6F0	UniProtKB (Swiss-Prot) 2026_03	248		gene name: TIP2-2; family: TIP
Oryza sativa	OsTIP3;1	Q9FWV6	UniProtKB (Swiss-Prot) 2026_03	264		gene name: TIP3-1; family: TIP
Oryza sativa	OsTIP3;2	Q7XKI5	UniProtKB (Swiss-Prot) 2026_03	265		gene name: TIP3-2; family: TIP
Oryza sativa	OsTIP4;1	Q75GA5	UniProtKB (Swiss-Prot) 2026_03	251		gene name: TIP4-1; family: TIP
Oryza sativa	OsTIP4;2	Q9LWR0	UniProtKB (Swiss-Prot) 2026_03	256		gene name: TIP4-2; family: TIP
Oryza sativa	OsTIP4;3	Q9LWR2	UniProtKB (Swiss-Prot) 2026_03	251		gene name: TIP4-3; family: TIP
Oryza sativa	OsTIP5;1	Q7XU31	UniProtKB (Swiss-Prot) 2026_03	269		gene name: TIP5;1; family: TIP
Oryza sativa	OsXIP	Q5WMW5	UniProtKB (Swiss-Prot) 2026_03	293		gene name: XIP; family: XIP
Helianthus annuus	HaNIP13	A0A251S9D7	UniProtKB (TrEMBL) 2026_03	300		gene name: NIP13; family: NIP
Helianthus annuus	HaNIP1;2	A0A251VQ75	UniProtKB (TrEMBL) 2026_03	276		gene name: NIP1:2; family: NIP
Helianthus annuus	HaNIP21	A0A251U880	UniProtKB (TrEMBL) 2026_03	284		gene name: NIP21; family: NIP
Helianthus annuus	HaNIP22	A0A251TKI2	UniProtKB (TrEMBL) 2026_03	280		gene name: NIP22; family: NIP

Species	Name used in this study (Figures 1, 2)	Accession number / gene identifier	Database / source	Length (aa)	Genomic location	Notes
Helianthus annuus	HaNIP6;1	A0A251US94	UniProtKB (TrEMBL) 2026_03	305		gene name: NIP6:1; family: NIP
Helianthus annuus	HaPIP1	A0A251T347	UniProtKB (TrEMBL) 2026_03	279		gene name: PIP1; family: PIP
Helianthus annuus	HaPIP12	A0A251UFS0	UniProtKB (TrEMBL) 2026_03	287		gene name: PIP12; family: PIP
Helianthus annuus	HaPIP1C	A0A251SVY1	UniProtKB (TrEMBL) 2026_03	244		gene name: PIP1C; family: PIP
Helianthus annuus	HaPIP21	A0A251UB90	UniProtKB (TrEMBL) 2026_03	284		gene name: PIP21; family: PIP
Helianthus annuus	HaPIP22	A0A251T1N0	UniProtKB (TrEMBL) 2026_03	284		gene name: PIP22; family: PIP
Helianthus annuus	HaPIP25	A0A251VE35	UniProtKB (TrEMBL) 2026_03	283		gene name: PIP25; family: PIP
Helianthus annuus	HaPIP2A	A0A251SJS2	UniProtKB (TrEMBL) 2026_03	283		gene name: PIP2A; family: PIP
Helianthus annuus	HaPIP2B	A0A251VPV4	UniProtKB (TrEMBL) 2026_03	289		gene name: PIP2B; family: PIP
Helianthus annuus	HaPIP2D	A0A251VPZ0	UniProtKB (TrEMBL) 2026_03	285		gene name: PIP2D; family: PIP
Helianthus annuus	HaPIP2E	A0A251T2N8	UniProtKB (TrEMBL) 2026_03	284		gene name: PIP2E; family: PIP
Helianthus annuus	HaPIP2F	A0A251TCY5	UniProtKB (TrEMBL) 2026_03	289		gene name: PIP2F; family: PIP
Helianthus annuus	HaPIP3B	A0A251RNF1	UniProtKB (TrEMBL) 2026_03	279		gene name: PIP3B; family: PIP

Species	Name used in this study (Figures 1, 2)	Accession number / gene identifier	Database / source	Length (aa)	Genomic location	Notes
Helianthus annuus	HaSIP12	A0A251VS51	UniProtKB (TrEMBL) 2026_03	237		gene name: SIP12; family: SIP
Helianthus annuus	HaSIP1;1	A0A251SR61	UniProtKB (TrEMBL) 2026_03	236		gene name: SIP1:1; family: SIP
Helianthus annuus	HaSIP2	A0A251U3C4	UniProtKB (TrEMBL) 2026_03	337		gene name: SIP2; family: SIP
Helianthus annuus	HaSIP2;1	A0A251TJR5	UniProtKB (TrEMBL) 2026_03	235		gene name: SIP2:1; family: SIP
Helianthus annuus	HaTIP	A0A251VAH2	UniProtKB (TrEMBL) 2026_03	250		gene name: TIP; family: TIP
Helianthus annuus	HaTIP11	A0A251VPN6	UniProtKB (TrEMBL) 2026_03	255		gene name: TIP11; family: TIP
Helianthus annuus	HaTIP12	A0A251ULJ2	UniProtKB (TrEMBL) 2026_03	252		gene name: TIP12; family: TIP
Helianthus annuus	HaTIP22	A0A251U692	UniProtKB (TrEMBL) 2026_03	248		gene name: TIP22; family: TIP
Helianthus annuus	HaTIP23	A0A251SW92	UniProtKB (TrEMBL) 2026_03	250		gene name: TIP23; family: TIP
Helianthus annuus	HaTIP31	A0A251SBP3	UniProtKB (TrEMBL) 2026_03	257		gene name: TIP31; family: TIP
Helianthus annuus	HaTIP4;1	A0A251SSD6	UniProtKB (TrEMBL) 2026_03	248		gene name: TIP4:1; family: TIP
Helianthus annuus	HaTIP5;1	A0A251SMU8	UniProtKB (TrEMBL) 2026_03	257		gene name: TIP5:1; family: TIP

Table S3. Analysis of the agronomic traits of wild-type and SiPIP1;5b-overexpressing tomato plants in the 2024 field trial, analysed according to the randomized complete block design. In this design the experimental unit is the plot (one plot per genotype in each of three replicate blocks), and the five plants measured per plot are subsamples. Because the plot is the experimental unit,

the primary inferential analysis is the comparison of plot means (n = 3 plots per genotype, each plot mean based on the five plants of that plot; two-tailed Student’s t-test), which is given in the first data column and used for the significance annotations in Figure 7. The two-way ANOVA of the plant-level data (n = 15 plants per genotype; 1 and 2 df for genotype and block, respectively, with 26 residual df), which accounts for the block structure, is given for comparison. The values shown in the main text and in Figure 7 are the means ± SEM of the 15 plants measured per genotype.

Trait	Plot-mean analysis P (n = 3; primary)	Genotype effect F(1,26)	P	Block effect F(2,26)	P
Plant height (cm)	0.040	9.68	0.0045	1.85	0.177
Stem diameter (mm)	0.891	0.01	0.909	0.12	0.888
Fruit shape index	0.0082	29.10	<0.0001	1.30	0.291
Fruit number per plant	0.041	12.32	0.0017	2.26	0.124
Individual fruit weight (g)	0.0007	24.88	<0.0001	0.08	0.927
